# Cigarette smoke components modulate the MR1–MAIT axis

**DOI:** 10.1084/jem.20240896

**Published:** 2025-01-17

**Authors:** Wael Awad, Jemma R. Mayall, Weijun Xu, Matt D. Johansen, Timothy Patton, Xin Yi Lim, Izabela Galvao, Lauren J. Howson, Alexandra C. Brown, Tatt Jhong Haw, Chantal Donovan, Shatarupa Das, Gesa J. Albers, Tsung-Yu Pai, Elinor Hortle, Caitlin M. Gillis, Nicole G. Hansbro, Jay C. Horvat, Ligong Liu, Jeffrey Y.W. Mak, James McCluskey, David P. Fairlie, Alexandra J. Corbett, Philip M. Hansbro, Jamie Rossjohn

**Affiliations:** 1 Infection and Immunity Program and Department of Biochemistry and Molecular Biology, Biomedicine Discovery Institute, Monash University, Clayton, Australia; 2 Immune Health Program, Hunter Medical Research Institute and The University of Newcastle, Newcastle, Australia; 3 ARC Centre of Excellence for Innovations in Peptide and Protein Science, Institute for Molecular Bioscience, University of Queensland, Brisbane, Australia; 4 Centre for Inflammation, Centenary Institute and University of Technology Sydney, Faculty of Science, School of Life Sciences, Sydney, Australia; 5 Department of Immunology and Microbiology, University of Melbourne at the Peter Doherty Institute for Infection and Immunity, Melbourne, Australia; 6 Centre for Innate Immunity and Infectious Diseases, Hudson Institute of Medical Research, Clayton, Australia; 7 School of Life Sciences, Faculty of Science, University of Technology Sydney, Ultimo, Australia; 8 Institute of Infection and Immunity, Cardiff University, School of Medicine, Cardiff, UK

## Abstract

Tobacco smoking is prevalent across the world and causes numerous diseases. Cigarette smoke (CS) compromises immunity, yet little is known of the components of CS that impact T cell function. MR1 is a ubiquitous molecule that presents bacterial metabolites to MAIT cells, which are highly abundant in the lungs. Using in silico, cellular, and biochemical approaches, we identified components of CS that bind MR1 and impact MR1 cell surface expression. Compounds, including nicotinaldehyde, phenylpropanoid, and benzaldehyde-related scaffolds, bound within the A′ pocket of MR1. CS inhibited MAIT cell activation, ex vivo, via TCR-dependent and TCR-independent mechanisms. Chronic CS exposure altered MAIT cell phenotype and function and attenuated MAIT cell responses to influenza A virus infection in vivo. MR1-deficient mice were partially protected from the development of chronic obstructive pulmonary disease (COPD) features that were associated with CS exposure. Thus, CS can impair MAIT cell function by diverse mechanisms, and potentially contribute to infection susceptibility and disease exacerbations.

## Teaser

Cigarette smoke is a diverse mix of chemicals that bind the antigen-presenting protein MR1 and modulate MAIT cell function in the lung through diverse mechanisms, impacting their ability to respond to respiratory infections and potentially contributing to disease.

## Introduction

MR1 is a highly conserved antigen-presenting molecule that is ubiquitously expressed by all nucleated human cells, with cell surface expression of MR1 being strongly ligand-dependent (Huang et al., 2005; McWilliam et al., 2016). MR1 can capture microbially derived metabolites formed during riboflavin biosynthesis, including the most potent mucosal-associated invariant T cell (MAIT) antigen, 5-(2-oxopropylideneamino)-6-D-ribitylaminouracil (5-OP-RU) (Corbett et al., 2014). Moreover, MR1 can present non-stimulatory folate-based ligands, such as 6-formylpterin (6-FP) and its derivative, acetyl-6-FP (Ac-6-FP), as well as drugs and drug-like molecules, diet-derived compounds, and endogenous host-derived ligands such as sulfated bile acids (Eckle et al., 2014; Ito et al., 2024; Keller et al., 2017; Kjer-Nielsen et al., 2012; Salio et al., 2020; Wang et al., 2022). Several MR1-binding ligands are anchored within the A′ pocket of MR1 by forming a Schiff base covalent bond with the MR1-Lys43 residue, triggering MR1 egress to the cell surface (Awad et al., 2020, 2023). However, the full spectrum of MR1-binding ligands remains to be determined.

Cigarette smoking is the third leading global cause of death worldwide. It is increasing in prevalence and drives numerous smoking-related lung and systemic pathologies, including chronic obstructive pulmonary disease (COPD), other respiratory and autoimmune diseases and cancer (Lee et al., 2012; Stämpfli and Anderson, 2009). There are no effective treatments for COPD due to an incomplete understanding of the pathogenic mechanisms. Cigarette smoke (CS) exposure adversely affects both innate and adaptive immunity (Le Rouzic et al., 2016; Mikko et al., 2013; Mortaz et al., 2009; Saint-André et al., 2024; Wu et al., 2021), including dysregulation of T cell activation (Beckett et al., 2013; Donovan et al., 2022; Hsu et al., 2017; Lambert et al., 2005). Defective immune responses occur in both active and passive (involuntary secondhand and thirdhand) smokers, as well as with exposure to other forms of smoke and air pollution, and effects may remain for years after cessation of exposure (Sleiman et al., 2010b; Wu et al., 2021). Defective immunity substantially increases susceptibility to respiratory infections that potently exacerbate the underlying disease. However, the mechanisms underlying these skewed immune responses and how they are related to smoke-associated diseases remain unclear.

CS is a complex mixture of thousands of xenobiotic chemicals resulting from the combustion, pyrolysis, and associated chemical reactions that occur during the burning of tobacco and other cigarette components (Rodgman and Perfetti, 2013). The chemical constituents of CS and their levels vary with tobacco type, blend, preparation, and additives, as well as the combustible portion of cigarettes, burning conditions, and other factors (Rodgman and Perfetti, 2013). Recently, it was shown that CS exposure reduced the ability of bronchial epithelial cells to stimulate MAIT cells in response to bacterial infection (Huber et al., 2023). Whether CS components can bind MR1 and/or modulate TCR-dependent MAIT cell effector functions is unclear. Through in silico, cellular, and structural approaches, we provide a molecular basis of how certain components of CS can bind MR1. We also show that CS components can inhibit MAIT cell activation. Furthermore, we show that chronic CS exposure dysregulates MAIT cells and their responses to infection in the lungs in vivo. These observations demonstrate that CS modulates the MR1–MAIT cell signaling axis, with implications for immune responses to respiratory infections and disease exacerbations.

## Results

### CS extract upregulates MR1 surface expression and alters MAIT cell activation

**Figure 1.**
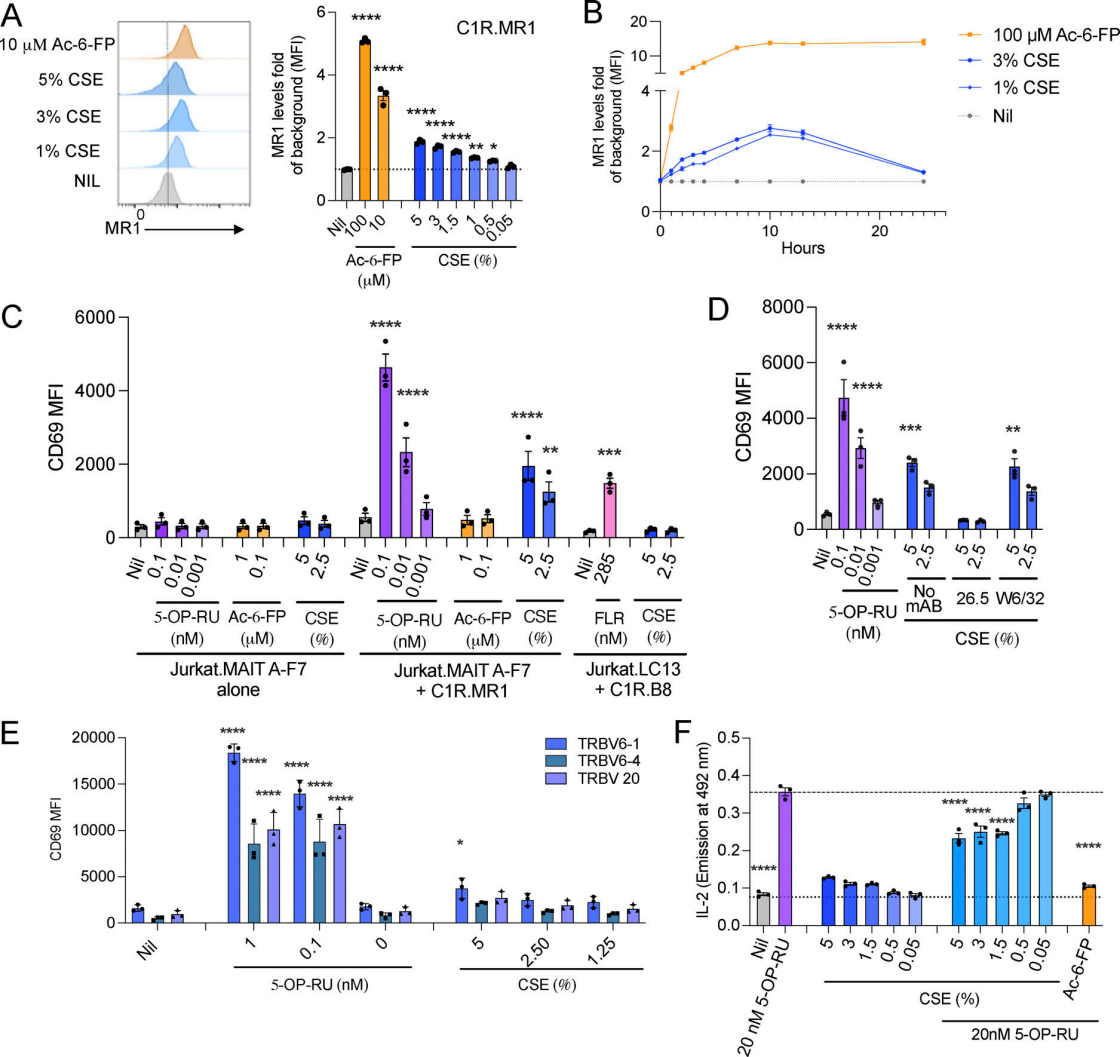
**Impact of CS extract on MR1 upregulation and MAIT reporter cell activation. (A)** Histogram and graph show surface expression of MR1 on C1R-MR1 cells in response to 3-h incubation with Ac-6-FP (100 µM or 10 µM) or CSE (5%–0.05 vol/vol%). **(B)** Time course shows dynamics of MR1 upregulation after adding Ac-6-FP (100 µM) or CSE (3% and 1%) to C1R.MR1 over 24 h. **(C)** CD69 expression in Jurkat.MAIT and Jurkat.LC13 (HLA-B8–EBV peptide-specific non-MAIT control) cells following activation with 5-OP-RU, Ac-6-FP, FLR, or CSE for 16-h co-culture with or without C1R.MR1 cells or with C1R.B8 as antigen-presenting cells. **(D)** Activation, detected by staining with anti-CD69, of Jurkat.MAIT cells after co-incubation with C1R.MR1 cells in the presence of 5-OP-RU or CSE, with or without anti-MR1 (26.5) or isotype control (W6/32) antibodies. The 26.5 and W6/32 antibodies were added to C1R.MR1 cells 2 h prior to co-incubation. **(E)** Activation of Jurkat.MAIT reporter cells expressing TRBV6-1, TRBV6-4, and TRBV20 TCRs, detected as CD69 expression, by 5-OP-RU, or CSE (5%, 2.5% and 1.25%) for 16 h in co-culture with C1R.MR1 as antigen-presenting cells. **(F)** Inhibition of Jurkat.MAIT reporter cell activation with 5-OP-RU by CSE. CSE was added to C1R.MR1 cells at the indicated concentrations and co-incubated with Jurkat.MAIT cells with or without 5-OP-RU. Data show emission at 492 nm, correlating with IL-2 production. **(A–F)** Data are shown as the mean ± SEM from three independent experiments. Statistical analysis by a one-way ANOVA followed by Dunnett’s multiple comparison test (* = P < 0.05, ** = P < 0.01, *** = P < 0.001, and **** = P < 0.0001).

**Figure S1.**
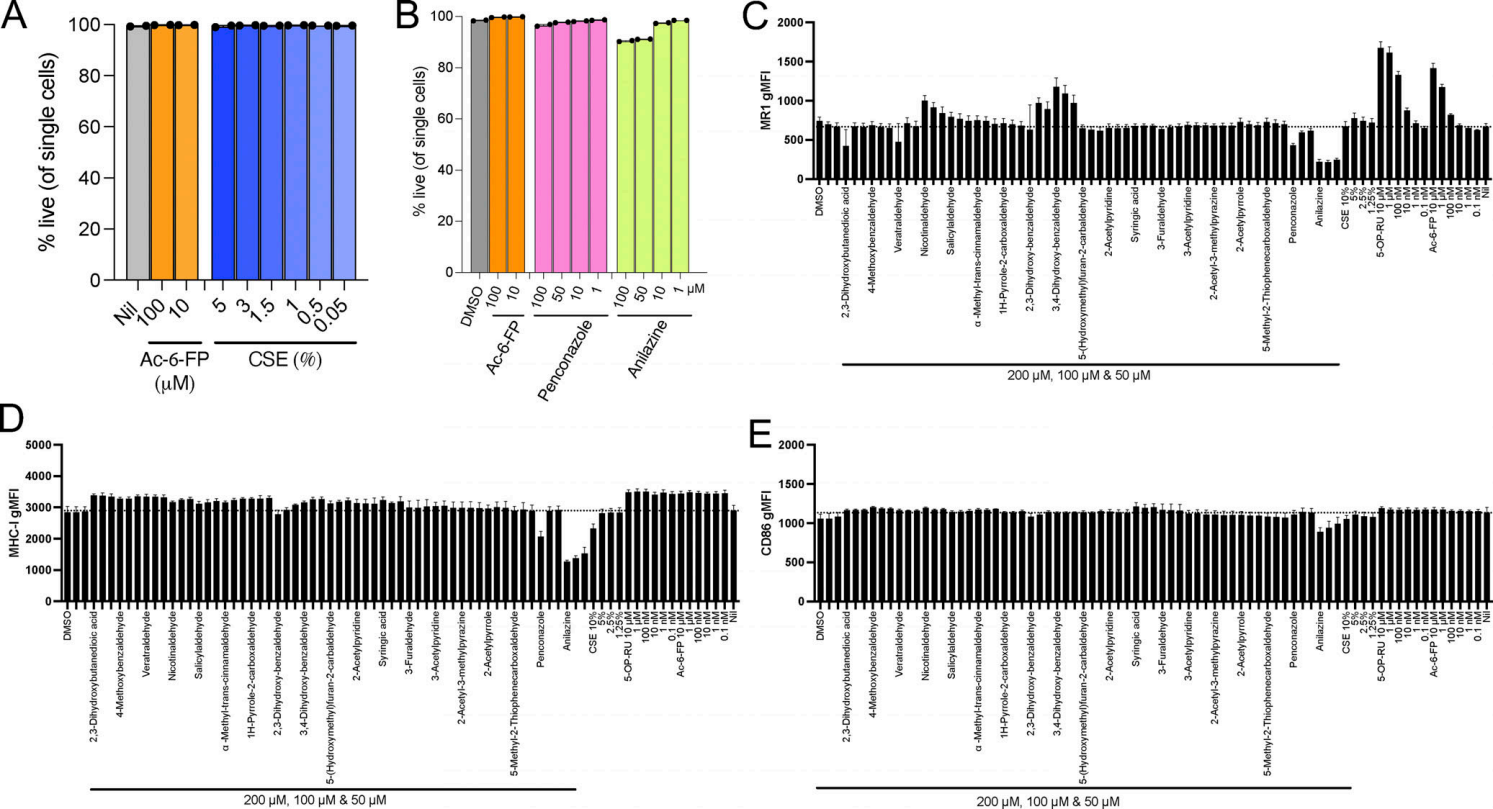
**Screening of compounds identified as CS components for antigen presentation. (A** **and B** **)** Proportion of cells in live cell gate after 3-h incubation with (A) Ac-6-FP (100–10 µM) or CSE (5–0.05), (B) DMSO, Ac-6-FP (100-10 µM), or 100-1 μM of indicated compounds. **(C–E)** Cell surface expression (geometric MFI [gMFI]) of MR1 (C), MHC I (D), and CD86 (E) on C1R cells in response to selected CS compounds or DMSO vehicle control, as labeled, at three concentrations (left to right 200, 100, and 50 μM), CSE (10%, 5%, 2.5%, and 1.25%), 5-OP-RU (10 μM to 0.1 nM, at 10-fold dilution), or Ac-6-FP (from 10 μM to 0.1 nM, at 10-fold dilution) for 3 h. The dotted line shows the gMFI value of Nil. Data are shown as the mean ± SEM from three independent experiments.

To address the impact of CS on MR1 presentation, we first examined the effects of soluble CS extract (CSE) on the cell surface expression of MR1 using the Class I–reduced (C1R) human lymphoblastoid cell line. CSE increased the expression of MR1 on the surface of C1R cells and C1R cells overexpressing MR1 (C1R.MR1) in a dose-dependent manner (0.05–5% vol/vol), albeit to a lesser extent than Ac-6-FP, a known potent upregulator of MR1 cell surface expression (Fig. 1 A and Fig. S1). The upregulation effect of MR1 with CSE was slower than that with Ac-6-FP and peaked at 10 h before declining, whereas it was prolonged for >24 h by Ac-6-FP, possibly due to differences in ligand stability (Fig. 1 B). No toxic effects on the cells were observed by live/dead staining when co-cultured with CSE concentrations of 5% or less (percent cell viability >95%) (Fig. S1 A).

Next, we explored whether CSE could activate Jurkat cells expressing MAIT TCRs: A-F7 (TRAV1-2/TRBV6-1), TRAV1-2/TRBV6-4, and TRAV1-2/TRBV20 when co-cultured with C1R.MR1 cells. A non-MAIT TCR-expressing cell line (Jurkat.LC13, which recognizes an EBV peptide FLRGRAYGL [FLR] bound to HLA-B8) co-cultured with C1R.B8 cells was used as control to report nonspecific effects. CSE-activated Jurkat.MAIT cells were measured by upregulation of CD69 and secretion of IL-2 (Fig. 1, C–F). There was no CD69 upregulation of Jurkat.LC13 cells when co-cultured with C1R.B8 cells or Jurkat.MAIT cells cultured without the C1R antigen-presenting cells (Fig. 1 C). As expected, in comparison to the extremely potent MAIT cell superagonist, 5-OP-RU, CSE activation of the MAIT TCR transductants was weaker, similar to other weak MAIT cell agonists previously described. Stimulation of Jurkat.MAIT cells by CSE was blocked in the presence of anti-MR1 (26.5) but not isotype control (anti-HLA, W6/32) monoclonal antibody, indicating that CSE-induced activation was MR1 dependent (Fig. 1 D). Activation was observed across all three Jurkat.MAIT cell lines tested (Fig. 1 E). CSE at higher doses moderately inhibited Jurkat.MAIT reporter cell production of IL-2 in response to 5-OP-RU (Fig. 1 F). Together, these data suggest that CSE causes upregulation of MR1 and can activate MAIT TCR-expressing T cell lines in an MR1-dependent manner.

### In silico prospecting of CS for MR1 ligands

**Figure 2.**
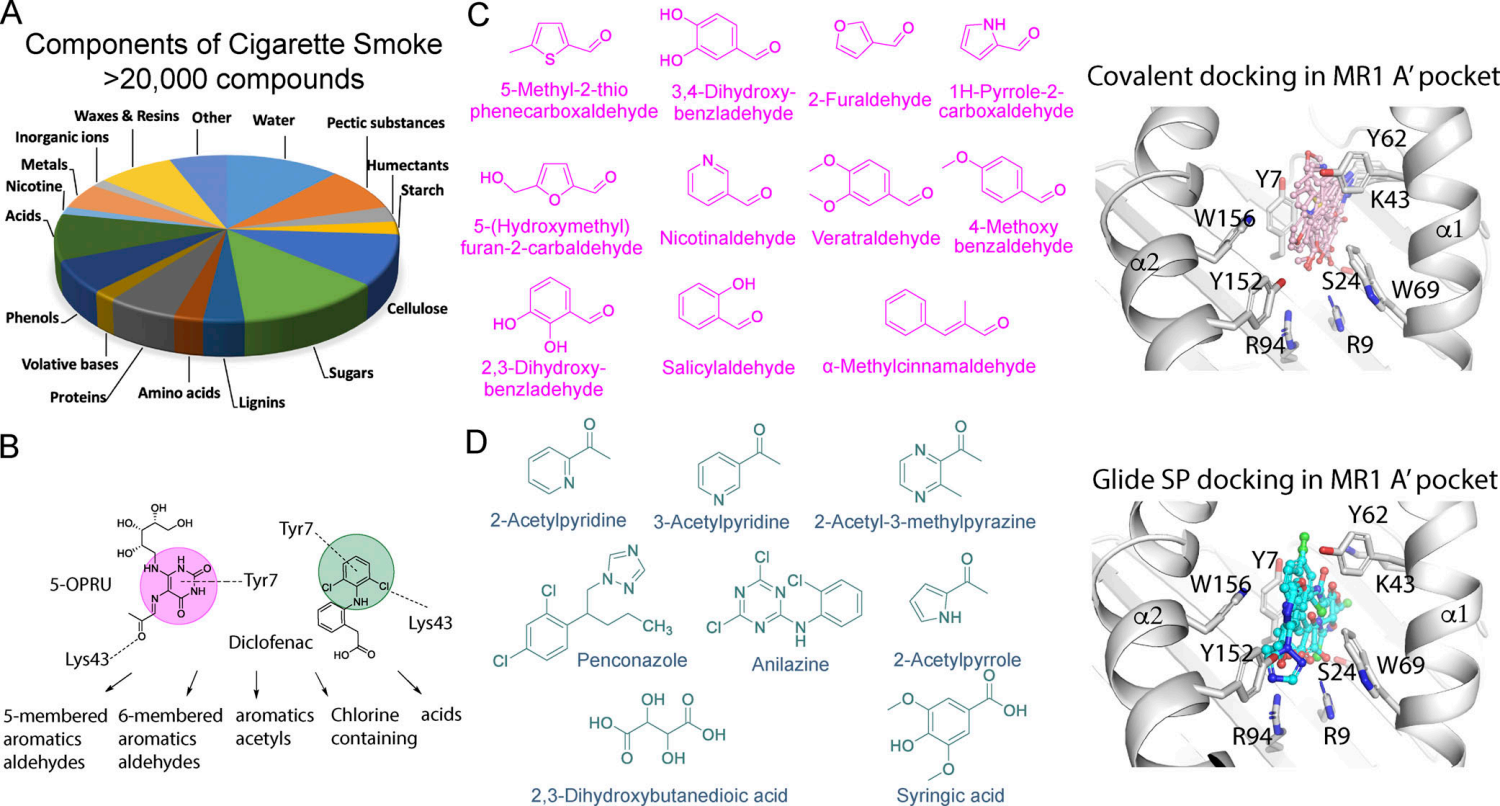
**In silico and molecular docking of the chemical components of CS as prospective ligands for MR1. (A)** CS is a complex mixture of products of tobacco combustion and other cigarette ingredients. **(B)** Prioritized compounds selected as putative ligands for MR1. **(C and D)** Chemical structures and molecular docking (using Glide) of CS components (pink) predicted to form a Schiff-based covalent bond with MR1-Lys43, including 5-membered and 6-membered aldehydes (C), or non-covalent bonds (cyan) within the putative ligand-binding cleft of MR1 (grey ribbon), including aromatic acetyls, chlorine-containing compounds, and acids (D). Key MR1 residues are shown as grey sticks and ligands are shown as ball and sticks (oxygen = red, nitrogen = blue, and chlorine = green).

CS is estimated to contain >20,000 heterogeneous and structurally diverse chemicals that can be inhaled (Fig. 2 A) (Rodgman and Perfetti, 2013). Aromatic aldehydes, ketones, and carboxylic acids account for ∼900 of these components (Bahl et al., 2016; Kosmider et al., 2016; Rodgman and Perfetti, 2013). To identify potential MR1-binding candidates in CS, we curated a chemical library of ∼6,000 organic compounds reported to form during the smoking process, followed by visual examination to identify 19 compounds that could potentially bind MR1 (Fig. 2 and Table S1). These included 5- and 6-membered ring aromatic aldehydes and acids, as well as chlorine-containing compounds hypothesized to bond to MR1 Lys43 in a similar manner to the MR1-binding ligand diclofenac (Keller et al., 2017) (Fig. 2 B). Covalent molecular docking simulations predicted 11 putative ligands that could form a Schiff base bond with Lys43 of MR1, each also possessing an aromatic ring that docked into the uracil-binding site like the potent MAIT cell antigen 5-OP-RU (Fig. 2 C). Another eight compounds were predicted to form diverse non-covalent interactions with other key MR1 residues (Fig. 2 D). These ligands are structurally dissimilar to 5-OP-RU and diclofenac (Fig. 2, C and D). In silico docking and chemoinformatic analysis suggested potential binding of these 19 structurally diverse ligands to the A′ pocket of MR1.

### Components of CS impact cell surface expression of MR1

**Figure 3.**
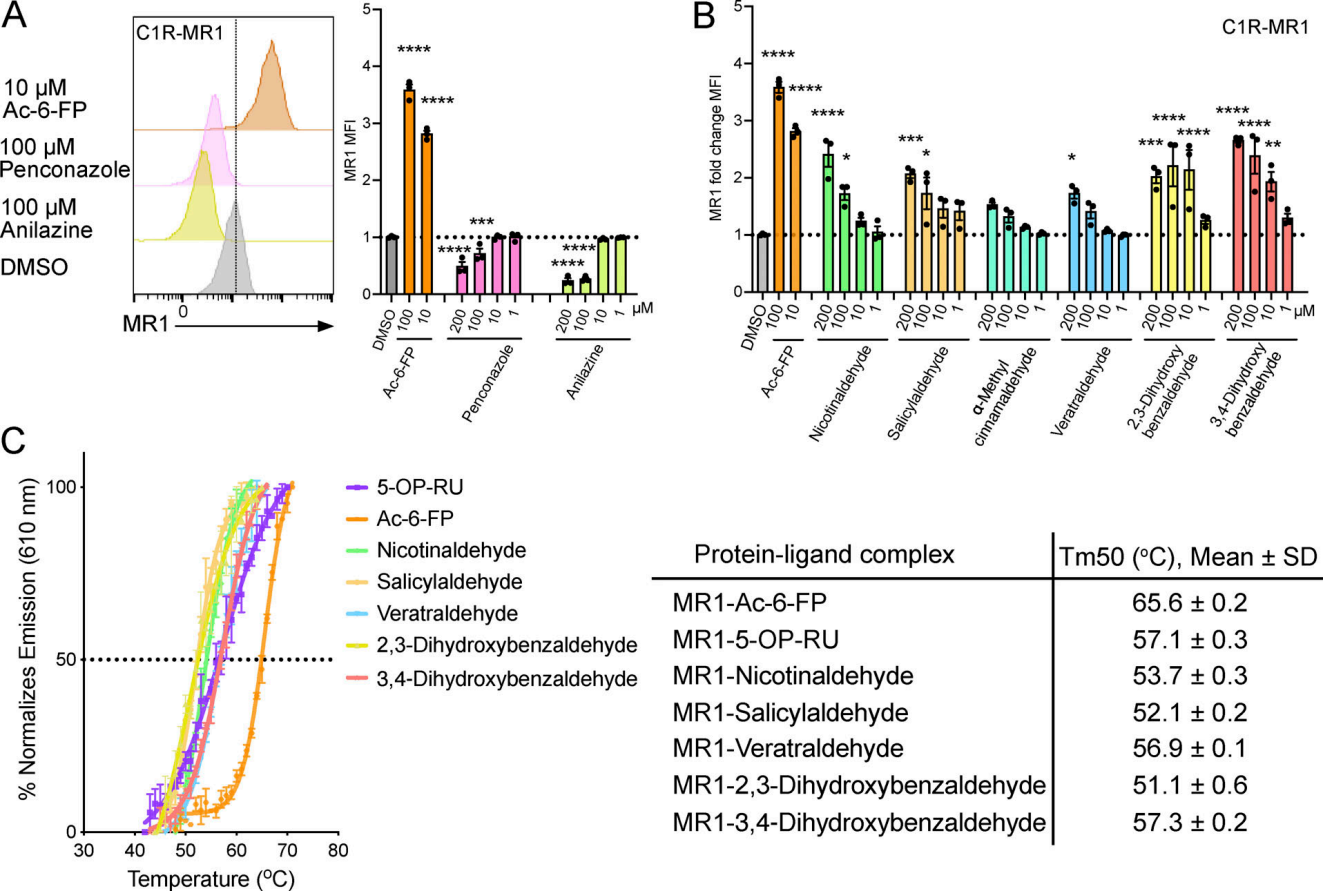
**CS components impact cell surface expression of MR1. (A)** Histogram and column graph showing cell surface expression of MR1 on C1R.MR1 cells (measured as MFI) in response to 3-h incubation with Ac-6-FP and penconazole and anilazine at the indicated doses. **(B)** Surface expression of MR1 on C1R.MR1 cells after 3-h incubation with Ac-6-FP, nicotinaldehyde, salicylaldehyde, α-methyl-trans-cinnamaldehyde, veratraldehyde, 2,3-dihydroxybenzaldehyde, or 3,4-dihydroxybenzaldehyde at indicated doses. DMSO was the vehicle control for all compounds. The dotted line shows the gMFI value of DMSO. Data show fold increases over background intensity (mean ± SEM from three independent experiments). Statistical analysis by a one-way ANOVA followed by Dunnett’s multiple comparison test (* = P < 0.05, ** = P < 0.01, *** = P < 0.001, and **** = P < 0.0001). **(C)** Thermostability of soluble MR1-CS ligands measured by fluorescence-based thermal shift assay. Graph shows baseline corrected, normalized emission at 610 nm plotted against temperature (°C) and Boltzmann curve fits. Each point represents the mean of three replicates, error bars represent SD. The Tm50 is the dotted line. The table summarizes the mean Tm50 across three independent experiments, each in triplicate.

The 19 CS-based compounds identified by in silico docking as candidate MR1-binding compounds were examined using in vitro functional assays (Fig. 3 and Fig. S1). Some aromatic ketones, aldehydes, and carboxylic acids predicted to dock into the MR1 A′ pocket (Fig. 2, B–D) did not affect the cell surface expression of MR1 (Fig. S1). This suggested that they either did not bind to MR1 or that binding within the MR1 pocket was insufficient to trigger MR1 cell surface expression as judged by anti-MR1 cell surface staining. Here, we identified eight compounds that did alter the cell surface expression level of MR1 (Fig. 3). Among these were two CS components, penconazole and anilazine, which are used as pesticides for tobacco plants, and they downregulated MR1 surface expression on C1R.MR1 and C1R cells after a short (3 h) incubation time (Fig. 3 A and Fig. S1). However, this downregulation was not specific to MR1 as both MHC-I and CD86 were also downregulated in C1R cells, albeit to a lesser extent for CD86 (Fig. S1). Although >90% of the cells were viable after 3–4 h of co-culture with penconazole and anilazine (Fig. S1 B), many of the cells died after overnight incubation. Next, we identified six CS candidates that upregulated MR1 on the cell surface of C1R and C1R-MR1 cells and did not increase MHC-I or CD86 levels (Fig. 3 B and Fig. S1). Their ability to upregulate MR1 was not as marked as that of Ac-6-FP, which is a potent ligand that induces MR1 cell surface expression. CS ligands upregulated MR1 included (1) benzaldehyde derivatives, such as salicylaldehyde, veratraldehyde, 2,3-dihydroxybenzaldehyde, and 3,4-dihydroxybenzaldehyde that are derived from complex phenolic components from tobacco during the smoking process, many of which are also added as flavorings to both conventional and e-cigarettes (Kosmider et al., 2016; Rodgman and Perfetti, 2013) (Table S1); (2) nicotinaldehyde, a pyrolysis product from tobacco present in third-hand smoke exposure and produced through the oxidation of nicotine deposited on indoor surfaces (Sleiman et al., 2010a); and (3) phenylpropanoid compounds, e.g., α-methyl-*trans*-cinnamaldehyde. Minimal cytotoxicity was observed for these compounds for the cells at the indicated concentrations and exposure times (cell viability >95%). These CS components upregulated MR1 on the C1R.MR1 cell surface in a dose-dependent manner over 3–4 h (Fig. 3 B), but higher MR1 expression was not observed after overnight incubation, possibly due to their volatile nature and consistent with the kinetics of MR1 upregulation by CSE (Fig. 1 B). Collectively, these results indicate that CSE contains a mixture of compounds that differentially affect MR1 surface expression, with the overall impact of CSE being to upregulate MR1 on the cell surface.

### Stability of CS components bound to MR1

To provide further evidence that CS components are ligands for MR1, we assessed whether they could mediate refolding of recombinant MR1 protein in solution. The CS-based compounds nicotinaldehyde, salicylaldehyde, veratraldehyde, 2,3-dihydroxybenzaldehyde, and 3,4-dihydroxybenzaldehyde all readily facilitated the refold of MR1 to enable downstream structural evaluation. To explore whether these CS components could affect the overall stability of MR1 protein in vitro, we performed thermostability assays on complexes of recombinant MR1 bound to individual CS ligands (MR1-CS) and compared the half-maximum melting temperatures (Tm50) to the values for MR1–5-OP-RU and MR1–Ac-6-FP complexes (Awad et al., 2020). MR1–CS complexes were stable at 37°C, and their Tm50 varied between 51°C and 57°C, indicating that the CS components stabilized the MR1 protein to a comparable extent as 5-OP-RU but to a lesser extent than Ac-6-FP (Fig. 3 C).

### Molecular basis of MR1 binding of CS components

**Figure 4.**
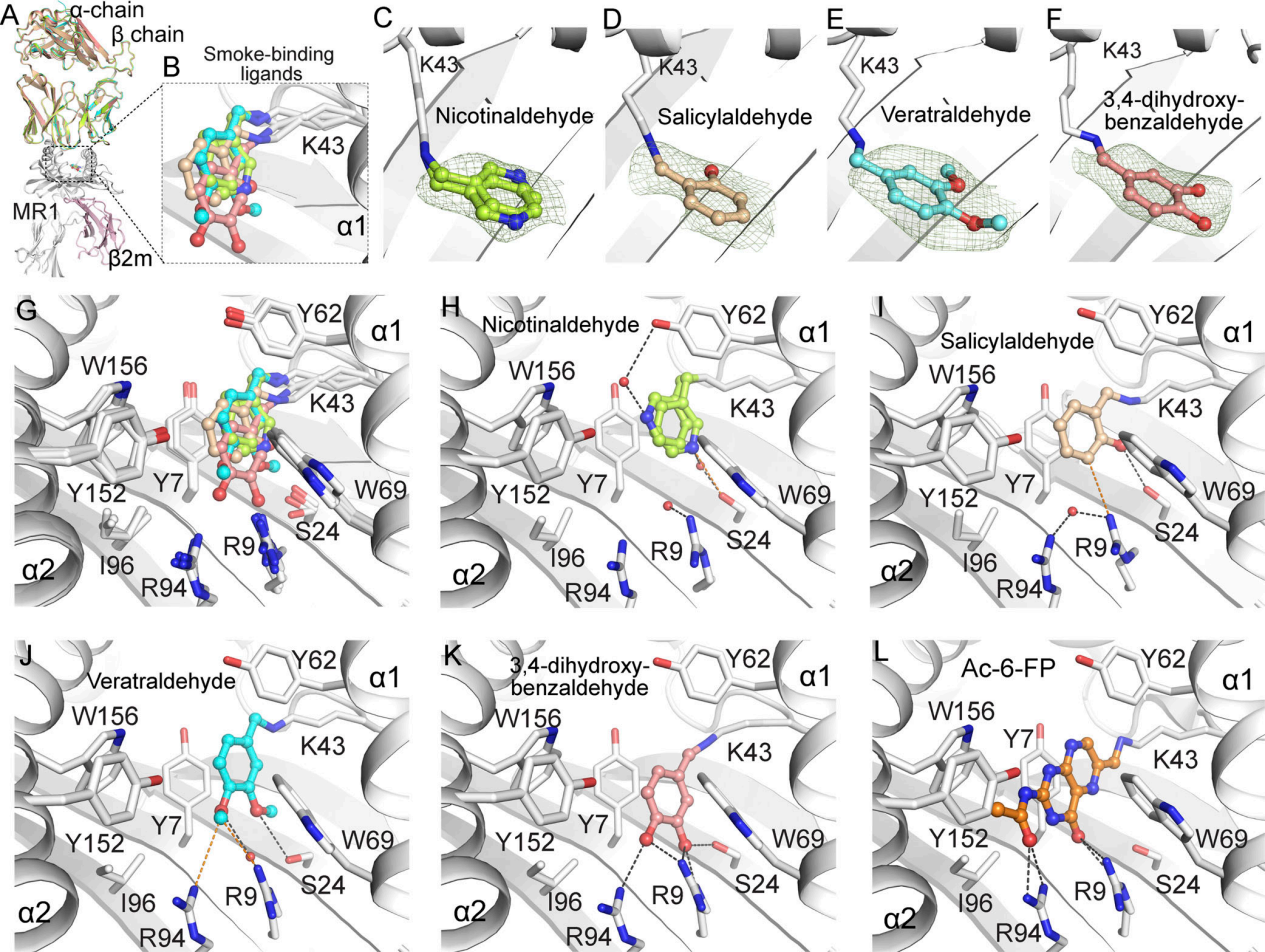
**Crystal structures of MR1 with ligands corresponding to CS components. (A and B)** Superposition of the ternary structures of TCR-MR1-CS–based ligands, with superposition of the CS-based ligands within the binding pocket shown as B. **(C–F)** The crystallographic unambiguous omit maps of nicotinaldehyde (C), salicylaldehyde (D), veratraldehyde (E), and 3,4-dihydroxybenzaldehyde (F) after simulated-annealing refinement (using the Phenix-refine crystallographic structure-refinement program), presented as an *F*_*o*_*− F*_*c*_ map (observed structure factor − calculated structure factor; smudge mesh) contoured at 3σ that highlight unambiguous positions of the ligands within the MR1 cleft. **(G)** Superposition of the MR1-binding ligands shows similar docking of the ligands within the A′ pocket of MR1. **(H–L)** Interactions between nicotinaldehyde (H), salicylaldehyde (I), veratraldehyde (J), 3,4-dihydroxybenzaldehyde (K), Ac-6-FP (PDB 4PJ5) (L), and the residues of MR1-A` portal in the MR1-Ag structures. All MR1-ligand interacting residues are shown as white sticks and water molecules are red spheres. Nicotinaldehyde, lemon; salicylaldehyde, wheat; veratraldehyde, cyan; 3,4-dihydroxybenzaldehyde, salmon; and Ac-6-FP, orange. Hydrogen bonding and van der Waals interactions are shown as black and orange dashed lines, respectively.

To understand the molecular basis for the binding and presentation of CS compounds to MR1, we determined crystal structures of MR1 presenting nicotinaldehyde, salicylaldehyde, veratraldehyde, or 2,3-dihydroxybenzaldehyde (Fig. 4 and Table S2). We used the MAIT A-F7 TCR as a crystallization aid analogous to the use of Fab fragments, as crystallization of MR1 binary structures is typically more challenging. Each of the four CS compounds was clearly visible within the A′ pocket of the MR1 antigen-binding cleft and each formed a Schiff base covalent bond with MR1-Lys43 in the pocket (Fig. 4, G–K). The aromatic rings of each CS compound adopted a similar plane to that observed for Ac-6-FP (Fig. 4, G–L); was wedged between MR1-Tyr7, Trp69, Tyr62, and Trp156 of the aromatic cradle of the MR1 pocket; and associated via van der Waals interactions (Fig. 4 G). Each of these ligands was located at the base of the pocket and did not interact with the key MR1-Tyr152 residue.

Specifically, nicotinaldehyde did not form any polar interactions within the pocket and formed two rotamers, showing its flexibility within the cleft and its propensity to form a water-bridged H-bond with either MR1-Tyr62 or MR1-Ser24 (Fig. 4 H). In contrast, all benzaldehyde analogues (salicylaldehyde, veratraldehyde, and 3,4-dihydroxybenzaldehyde) were stabilized inside the MR1 cleft through forming a direct H-bond between their carbonyl group and MR1-Ser24 at the base of the pocket (Fig. 4, H–J). Here, the salicylaldehyde ligand did not exhibit further polar interactions within the pocket (Fig. 4 I), yet the veratraldehyde formed an additional water-bridged H-bond with MR1-Arg9 and aromatic interactions with Arg9 and Arg94 at the base of the MR1 cleft (Fig. 4 J). The 3,4-dihydroxybenzaldehyde ligand formed a stronger network of H-bonds with MR1-Arg9 and Arg94 that resulted in a small displacement (∼1.5 Å) of its ring deeper into the pocket compared with the other CS components (Fig. 4, G and K). These additional interactions are consistent with the finding that 3,4-dihydroxybenzaldehyde is a relatively stronger cell surface upregulator of MR1 compared with other CS ligands (Fig. 3 B). Notably, these four CS ligands, while making numerous contacts with MR1, did not contact the A-F7 MAIT TCR, suggesting that these ligands would not activate MAIT cells.

### MR1-binding CS components can inhibit MAIT cell activation in vitro

**Figure 5.**
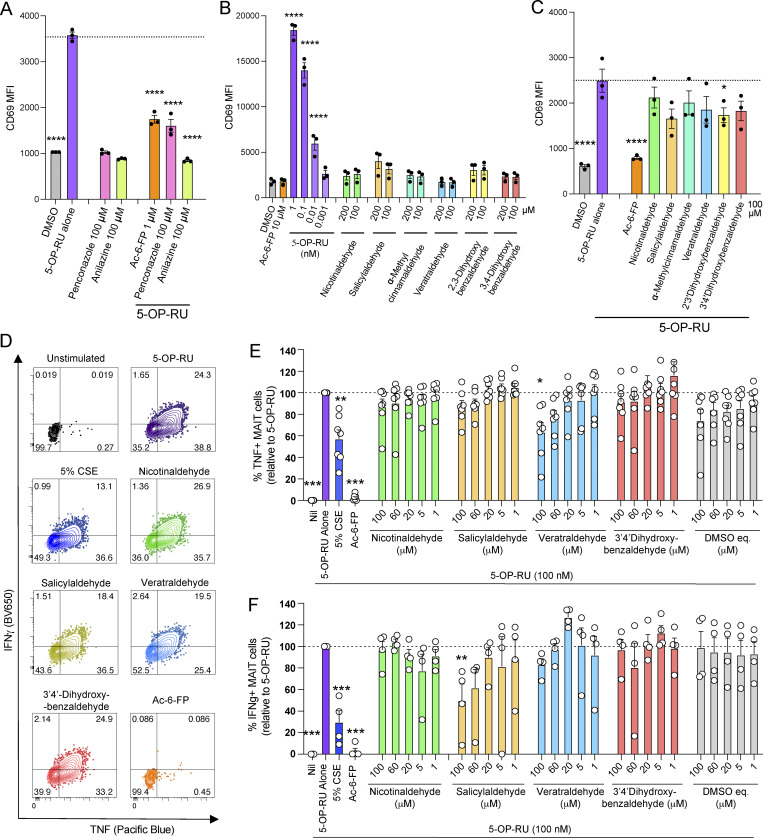
**Effects of CS extract and its components on and MAIT cells and non-MAIT T cells. (A)** Activation of Jurkat.MAIT AF-7 cells detected as CD69 expression, by penconazole or anilazine alone (100 μM) or in the presence of 0.01 nM 5-OP-RU, after 3-h co-culture with C1R.MR1 antigen-presenting cells. **(B and C)** Activation of Jurkat.MAIT AF-7 reporter cells, detected as CD69 expression, by indicated compounds alone (200 and 100 μM) (B) or in the presence of 0.01 nM 5-OP-RU (C), after overnight (∼16 h) co-culture with C1R.MR1 antigen-presenting cells. Data show mean ± SEM from three independent experiments. Statistical analysis by a one-way ANOVA followed by Dunnett’s multiple comparison test (* = P <0.05 and **** = P < 0.0001). **(D–F)** PBMCs were incubated for 6 h with 5-OP-RU (0.1 nM) ±5% CSE at a range of concentrations or CS compounds veratraldehyde, nicotinaldehyde, salicylaldehyde, or 3,4-dihydroxybenzaldehyde (100, 60, 20, 5, and 1 μM) or Ac-6-FP (100, 60, 20, 5, and 1 μM); including 5-h inhibition of cytokine secretion with Golgi plug (mean ± SEM, two independent experiments). Cells were stained intracellularly for cytokines IFNγ (four donors) and TNF (seven donors) and analyzed by flow cytometry to determine the impact on MAIT cell activation. Equivalent doses of DMSO were used as the vector control for the compounds. For gating strategy and % live cell data, see Fig. S5. **(D)** Contour plot shows intracellular staining profile of TNF and IFNγ by MAIT cells. **(E and F)** Plot shows the total percentage of MAIT cells positive for TNF (E) or for IFNγ (F) in response to 5-OP-RU in the presence or absence of CSE (5%, 2%, 1.25%, and 0.73%), 100 μM of indicated compounds or Ac-6-FP, normalized to the 5-OP-RU control. Statistical analysis by a one-way ANOVA with Holm–Sidak post hoc test (* = P < 0.05, ** = P < 0.01, and *** = P < 0.001).

**Figure S2.**
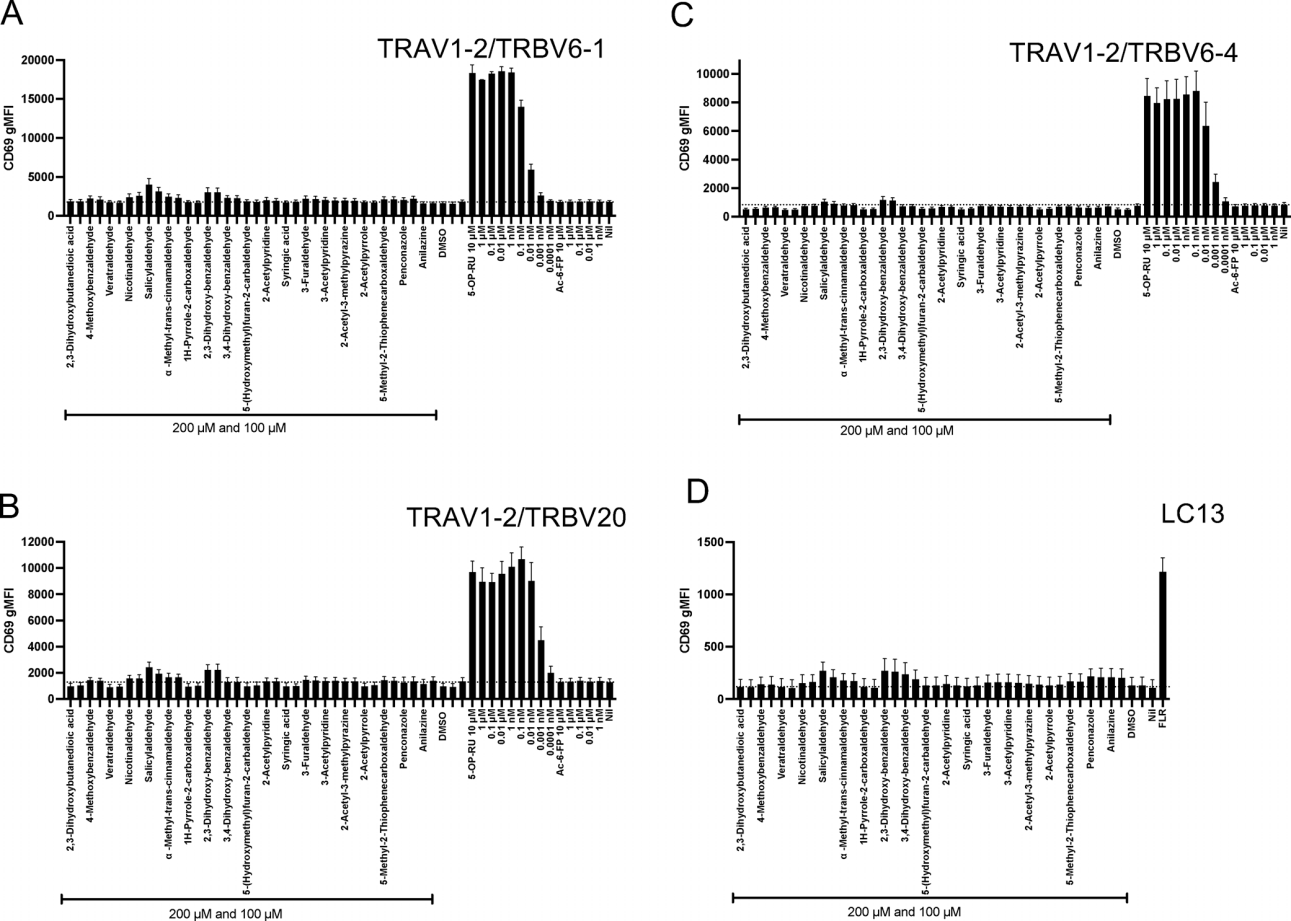
**Jurkat.MAIT activation by smoke compounds**. **(A–D)** Activation of Jurkat.MAIT reporter cells expressing TRBV6-1 (A), TRBV6-4 (B), TRBV20 (C), or Jurkat.LC13 (HLA-B8–EBV peptide-specific non-MAIT control) (D), detected as CD69 expression following incubation with 5-OP-RU (from 10 μM to 0.0001 nM, at 10-fold dilution) (A–C), Ac-6-FP (from 10 μM to 1 nM, at 10-fold dilution) (A–C), or smoke candidate compounds (200 and 100 μM), for 16 h in co-culture with C1R.MR1 cells (A–C) or C1R.B8 (D) as antigen-presenting cells. FLR peptide gives maximal activation of Jurkat.LC13. Data are shown as the mean ± SEM from three independent experiments.

We then evaluated whether these CS-based compounds could modulate the activation of Jurkat.MAIT reporter cells. Consistent with the structural data, none of the investigated 19 CS candidates induced substantial CD69 upregulation by the three Jurkat.MAIT cell lines assessed compared with the Jurkat.LC13 control (Fig. 5, A–C and Fig. S2). Next, a subset of CS ligands, which we demonstrated can impact MR1 surface expression, were examined for their ability to competitively inhibit MR1-dependent Jurkat.MAIT cell activation by 5-OP-RU. Penconazole and anilazine (100 µM concentrations) inhibited the 5-OP-RU–dependent activation of the Jurkat.MAIT.A-F7 cells, as measured by surface expression of the activation marker CD69 after a 3-h incubation (cell viability ∼95%) (Fig. 5 A). We found that the CS-based ligands that upregulated MR1 expression were also weak inhibitors of activation, when tested in the Jurkat.MAIT co-culture system (Fig. 5 C). The efficacy of these compounds in inhibiting TCR-dependent MAIT cell activation approximately correlated with their efficacy in upregulating cell surface MR1 (Fig. 3 B and Fig. 5 C). These CS compounds, including nicotinaldehyde, phenylpropanoid, and benzaldehyde derivatives, did not alter MHC-I or CD86 expression on C1R cells (Fig. S1), suggesting a specific inhibitory effect on the MR1–MAIT TCR axis.

### CSE and MR1-binding CS components inhibit MAIT cell effector functions ex vivo

**Figure S3.**
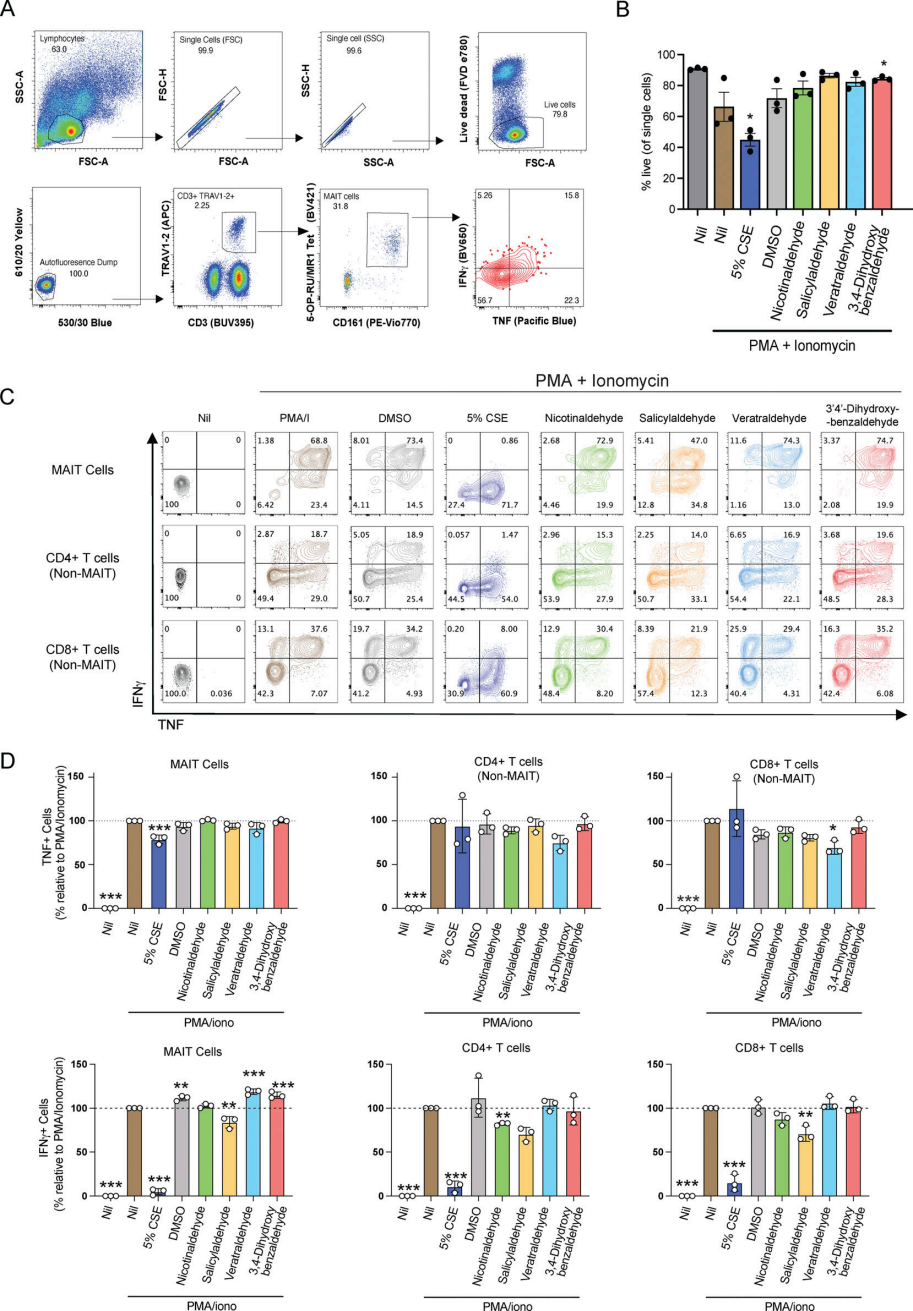
**Activation/inhibition of MAIT cells and T cells within PBMCs by CSE, CS, and components. (A)** Gating strategy for MAIT cells, which were defined as CD3^+^ MR1–5-OP-RU tetramer^+^ TRAV1-2^+^ CD161^++^ live lymphocytes. PBMCs were incubated for 6 h with 5-OP-RU (0.1 nM) ± CSE (5%); Ac-6-FP (100, 60, 40, 20, 5, and 1 μM); or the indicated CS compounds (100, 60, 40, 20, 5, and 1 μM), including 5-h inhibition of cytokine secretion with Golgi plug. Cells were stained intracellularly for cytokines analyzed by flow cytometry. **(B–D)** PBMCs were activated with PMA (5 ng/ml) and ionomycin (1 μg/ml) following 1-h incubation in the presence or absence 5% CSE or 100 μM of indicated compounds. BD Golgi plug was added and cells incubated for 18 h, before being stained with surface antibodies to identify MAIT, CD4^+^ (non-MAIT), and CD8^+^ (non-MAIT) T cells, stained intracellularly with antibodies to cytokines (TNF-APC and IFNγ-AF700) and analyzed by flow cytometry. **(B)** Proportion of cells in the live cell gate. **(C)** Representative plots showing the expression of cytokines IFNγ and TNF by MAIT and CD4^+^ and CD8^+^ T cell subsets. **(D)** Graphs summarize the proportion of indicated T cell subsets producing cytokines (mean ± SEM of three donors). Statistical analysis by a one-way ANOVA with Holm-Sidak post hoc test (* = P < 0.05, ** = P < 0.01, *** = P < 0.001).

To examine the impact of CSE and CS compounds on circulating MAIT cells, human peripheral blood mononuclear cells (PBMCs) from healthy donors were cultured for 6 h with 5-OP-RU with and without prior exposure to CSE or CS compounds veratraldehyde, 3,4-dihydroxybenzaldehyde, nicotinaldehyde, or salicylaldehyde, or Ac-6-FP as a known inhibitor (Fig. 5, D–F and Fig. S3). MAIT cells were identified as TRAV1-2^+^ MR1–5-OP-RU tetramer^+^ CD161^+^ CD3^+^ lymphocytes, and the expression of intracellular TNF and IFN-γ was measured by flow cytometry (Fig. S3 A). We did not observe toxicity by live/dead staining of PBMCs cultured with the indicated CSE concentrations and/or 5-OP-RU (>80% live cells). CSE reduced the resultant MAIT cell production of TNF and IFN-γ in response to 5-OP-RU. Individual veratraldehyde and salicylaldehyde compounds showed some inhibition of MAIT cell activation by 5-OP-RU, as observed by a decrease in the production of TNF and IFN-γ, respectively (Fig. 5, D–F).

**Figure S4.**
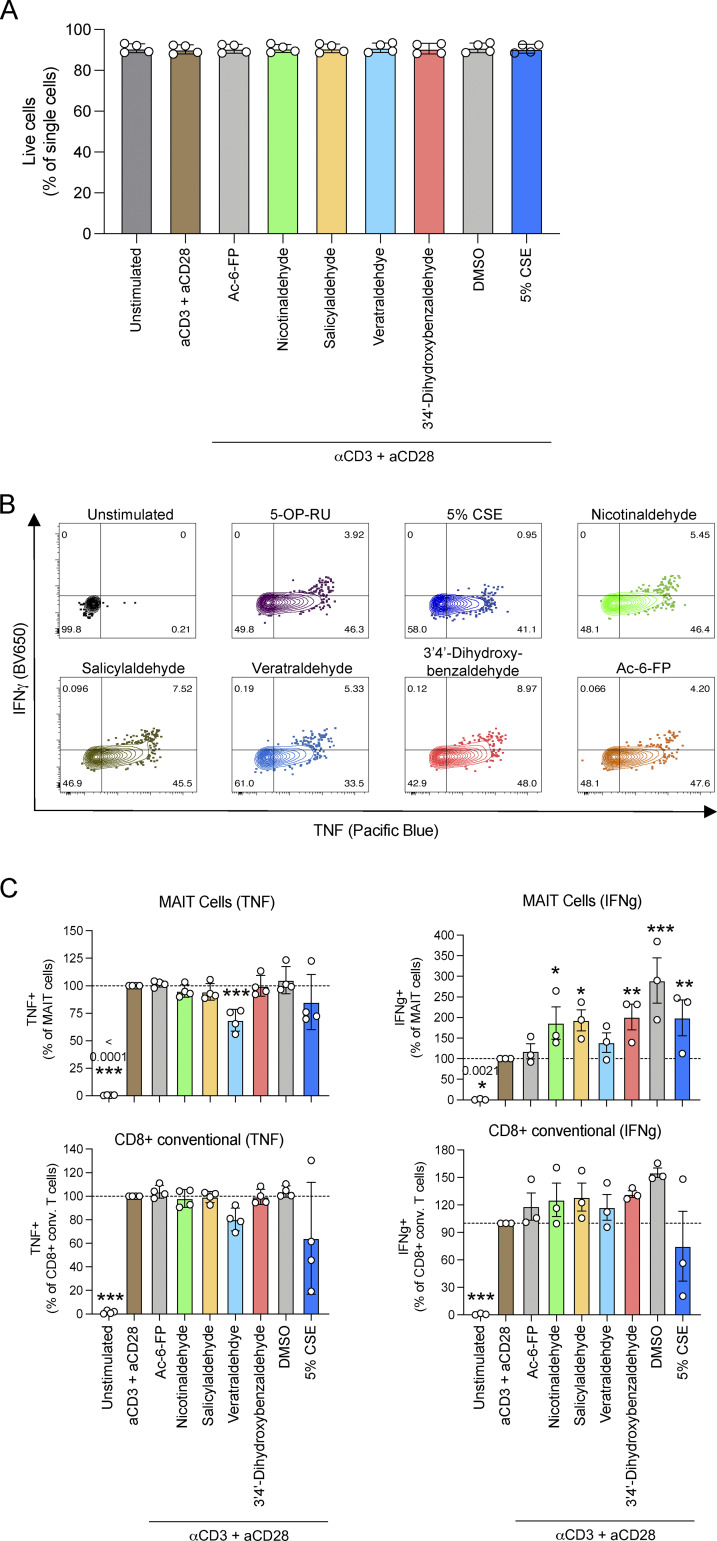
**Effect of CSE and components on TCR independent (CD3/CD28) activation of T cells. (A–C)** PBMCs were activated for 6 h with plate-bound α-CD3 (#555 329; 10 μg/ml; BD) and α-CD28 (#555 729; 2 μg/ml; BD) following 1-h incubation in the presence or absence 5% CSE or 100 μM of indicated compounds. BD Golgi plug was added and cells for the last 5 h of stimulation. Cells were then stained with surface antibodies to identify MAIT and CD4^+^ (non-MAIT) and CD8^+^ (non-MAIT) T cells, stained intracellularly with antibodies to cytokines (TNF-Pacific blue and IFNγ-BV650) and analyzed by flow cytometry. **(A)** The proportion of cells in the live cell gate. **(B)** Representative plots showing the expression of cytokines IFNγ and TNF by MAIT and CD4^+^ and CD8^+^ T cell subsets. **(C)** Plots showing the percentage of MAIT or CD8^+^ conventional T cells positive for either TNF or IFNγ, in response to CD3/CD28 stimulation in the presence or absence of CSE (5%), 100 μM of indicated compounds or Ac-6-FP; normalized to the CD3/CD28 control (% positive). Graphs show the mean ± SEM of three to four donors from one independent experiment. Statistical analysis by a one-way ANOVA with Holm-Sidak post hoc test (* = P < 0.05, ** = P < 0.01, *** = P < 0.001).

It has been reported previously that exposure to CS can inhibit IFN-γ production by conventional T cells in a TCR-independent manner by reducing the recruitment of positive IFN-γ transcriptional regulators (Feng et al., 2011). Therefore, we next investigated whether MAIT cell effector functions would be similarly inhibited in a TCR-independent manner by CSE. We found that co-culturing PBMCs with CSE reduced IFN-γ production by MAIT cells, with similar effects on conventional CD4 and CD8 T cell subsets, in response to TCR-independent activation by phorbol-12-myristate-13-acetate (PMA) and ionomycin (Fig. S3, C and D). However, CSE did not inhibit TNF cytokine production by non-MAIT T cell subsets upon PMA/ionomycin activation, whereas there was a small reduction for MAIT cells. (Fig. S3, C and D). Notably, the viability of CD3^+^ cells decreased when PBMCs were co-cultured with PMA/ionomycin and 5% CSE (∼50% live cells), compared with PMA/ionomycin alone cultured cells (∼70% live cells), indicating that toxicity may impact cellular signaling and effector function (Fig. S3 B). We next tested a milder stimulation of cells using anti-CD3 and anti-CD28, which did not substantially affect cell viability (Fig. S4). In this antigen-independent setting, mild inhibitory effects were observed for both MAIT and CD8^+^ non-MAIT T cells by CSE and veratraldehyde, but not by salicylaldehyde (Fig. S4, B and C). Together, these results indicate that there are both MR1-specific and nonspecific inhibitory effects of CSE and CS compounds on MAIT cell activation.

### CS exposure alters MAIT cell responses and phenotype in vivo

**Figure S5.**
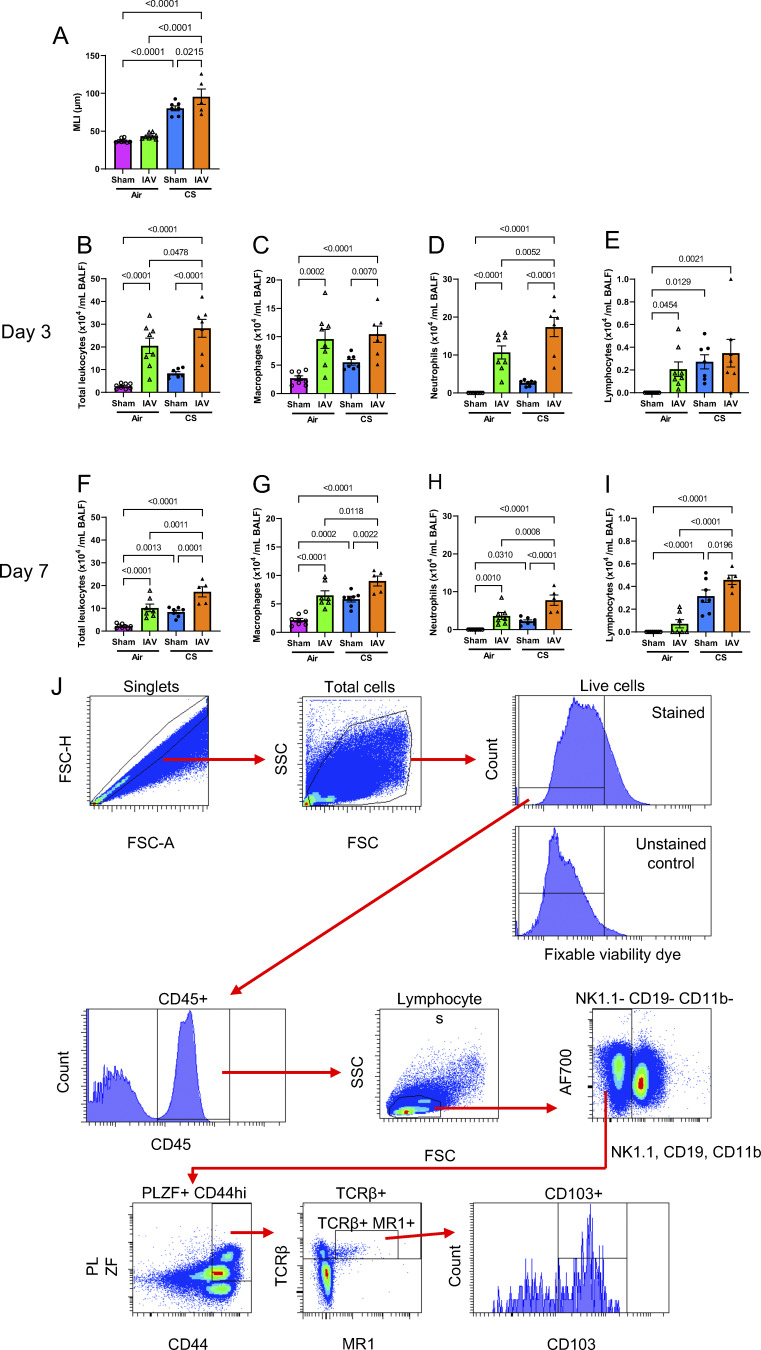
**Airway inflammation during in vivo CS exposure and IAV infection, and gating strategy for characterization of murine lung MAIT cells. (A–I)** Alveolar diameter as measured using the alveolar wall MLI technique in lung tissue sections and (B and F) total leukocytes, (C and G) macrophages, (D and H) neutrophils, and (E and I) lymphocytes in BALF from mice exposed to normal air or CS for 10 wk, followed by inoculation with IAV or sham inoculation, assessed at day 3 (B–E) or 7 (A and F–I) after inoculation. All data are presented as mean ± SEM. *N* = 5–8 mice. Statistical analysis by one-way ANOVA with Fisher’s least significant difference post hoc test. **(J)** Gating strategy for the identification and characterization of MAIT cells (CD45^+^ TCRβ^+^ MR1–5-OP-RU tetramer^+^ PLZF^+^ CD44^hi^ NK1.1^−^ CD19^−^ CD11b^−^) in single-cell suspensions of the mouse lung tissue. Doublets, debris, and dead cells were first excluded. Leukocytes (CD45^+^ cells) were then selected, followed by lymphocytes (forward scatter [FSC]^lo-int^ side scatter [SSC]^lo^). FITC was used as a dump channel to exclude NK1.1^+^, CD19^+^, and CD11b^+^ cells. Following this, PLZF^+^ CD44^hi^ cells were selected and then finally TCRβ^+^ MR1–5-OP-RU tetramer^+^ MAIT cells were identified. MR1 reactivity was determined by comparison of MR1–5-OP-RU tetramer staining to 6-FP control tetramer staining. MAIT cells were further characterized as CD103^+/−^.

The above data showed that CS exposure inhibited ex vivo activation of MAIT cells from healthy donor PBMCs (Fig. 5), yet how smoking directly impacts MAIT cell function in vivo in the lungs is less clear. To address this, C57BL/6 mice were exposed to either CS or normal air for 2–12 wk. Some groups of mice smoked for 10 wk were subsequently challenged with influenza A virus (IAV) or vehicle alone (sham) after CS exposure, and the lungs assessed at 3 and 7 days postinfection (dpi) (Fig. S5). By 10 wk, CS-exposed mice exhibited lung disease with airway inflammation and emphysema-like alveolar enlargement that was exacerbated by IAV infection (Fig. S5). This is consistent with the presentation and exacerbation of CS-induced COPD in humans (Beckett et al., 2013; Hsu et al., 2015, 2017; Kwon et al., 2016).

**Figure 6.**
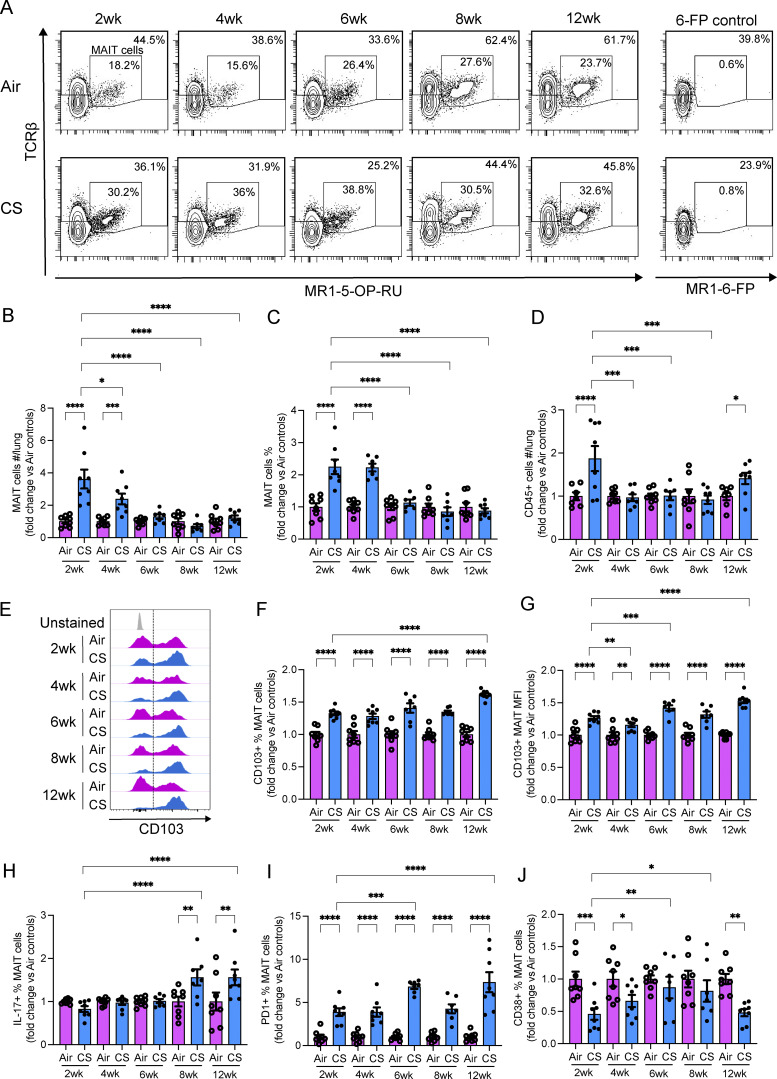
**CS exposure alters MAIT cell numbers and functional marker expression in vivo. (A)** Representative flow cytometry plots, including MAIT cells (CD45^+^ TCRβ^+^ MR1–5-OP-RU tetramer^+^ PLZF^+^ CD44^hi^ NK1.1^−^ CD19^−^ CD11b^−^), in lung homogenates from mice exposed to normal air or CS for 2, 4, 6, 8, or 12 wk. **(B–D)** (B) Total numbers of MAIT cells per lung, (C) frequency of MAIT cells as a percentage of CD45^+^ cells, and (D) total numbers of CD45^+^ cells per lung, in lung homogenates from mice as in A. **(E–J)** Representative flow cytometry plots showing (E) CD103 expression on MAIT cells, (F) frequency of CD103^+^ MAIT cells as a percentage of total MAIT cells, and (G) MFI of CD103 staining on MAIT cells, in lung homogenates from mice as in A. Frequency of (H) IL-17^+^, (I) PD1^+^, and (J) CD38^+^ MAIT cells as a percentage of total MAIT cells, in lung homogenates from mice as in A. All data expressed as fold change to air exposed controls and presented as mean ± SEM. *N* = 7–8. Statistical analysis by one-way ANOVA with Fisher’s least significant difference post hoc test (* = P < 0.05, ** = P < 0.01, *** = P < 0.001, and **** = P < 0.0001).

Total MAIT cell numbers and the frequency of MAIT cells as a proportion of CD45^+^ cells were increased in the lungs of mice at both 2 (P < 0.0001) and 4 (total numbers: P = 0.0002; frequency: P < 0.0001) wk of CS exposure, compared with air-exposed controls (Fig. 6, A–C). The total numbers of CD45^+^ cells in the lungs were increased at both 2 (P < 0.0001) and 12 (P = 0.0379) wk of CS exposure (Fig. 6 D).

**Figure 7.**
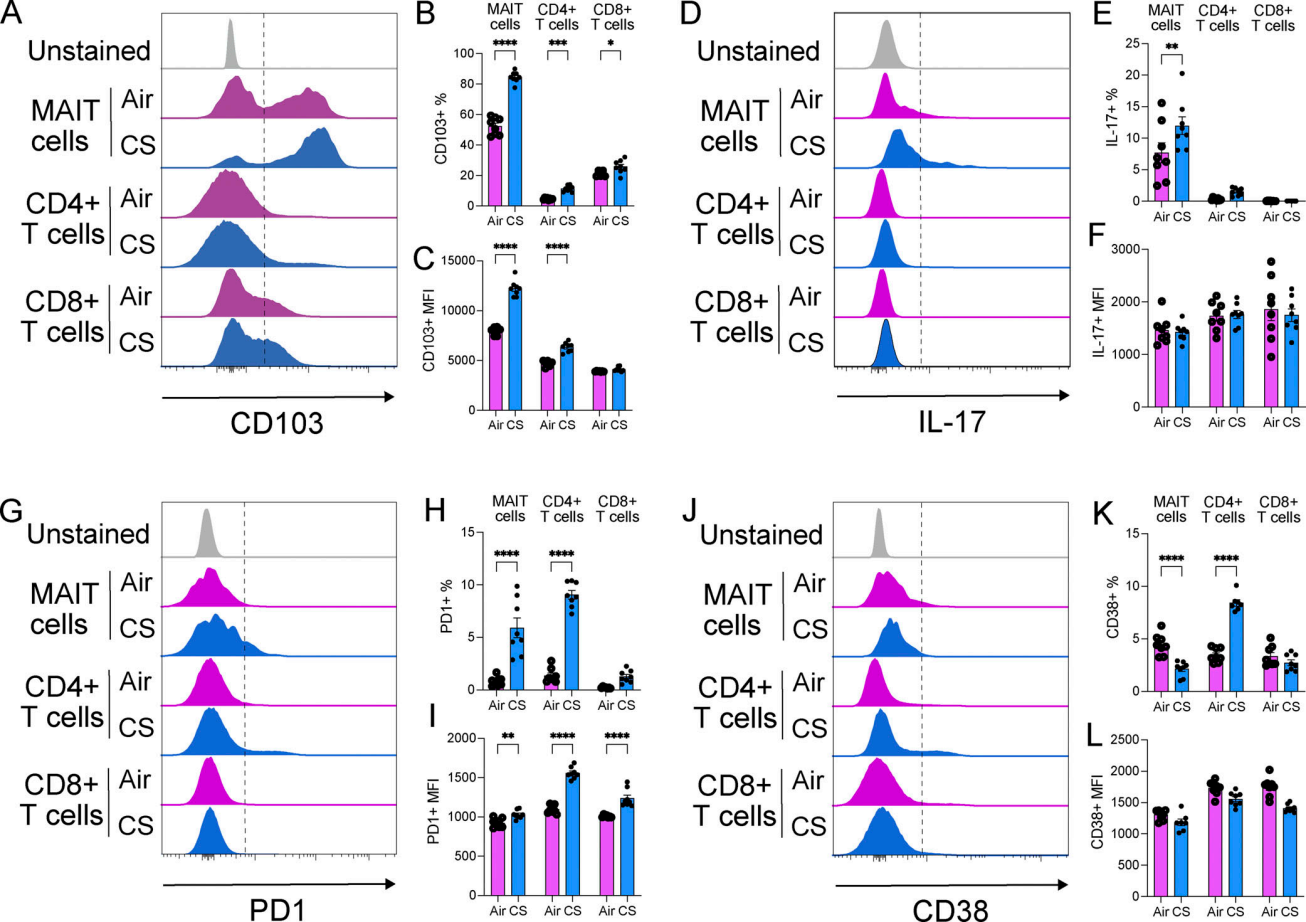
**CS exposure differentially alters CD103, IL-17, PD1, CD38 expression in MAIT, CD4** ^**+**^ **, and CD8** ^**+**^ **T cells. (A–L)** Representative flow cytometry histograms comparing (A) CD103, (D) IL-17, (G) PD1, or (J) CD38 expression on MAIT cells (CD45^+^ TCRβ^+^ MR1–5-OP-RU tetramer^+^ PLZF^+^ CD44^hi^ NK1.1^−^ CD19^−^ CD11b^−^), as shown in Fig. 6, compared with CD4^+^ and CD8^+^ T cells (CD45^+^ TCRβ^+^); frequency of (B) CD103^+^, (E) IL-17^+^, (H) PD1^+^, or (K) CD38^+^ cells as a percentage of total MAIT and CD4^+^ and CD8^+^ T cells; MFI of positive cells for (C) CD103, (F) IL-17, (I) PD1, or (L) CD38 staining on MAIT and CD4^+^ and CD8^+^ T cells; in lung homogenates from mice exposed to normal air or CS for 12 wk from experiment as in Fig. 6. All data presented as mean ± SEM. *N* = 7–8 mice. Statistical analysis by one-way ANOVA with Fisher’s least significant difference post hoc test (* = P < 0.05, ** = P < 0.01, *** = P < 0.001, and **** = P < 0.0001).

The MAIT cell phenotype was also altered by CS exposure in vivo, with both the frequency and mean fluorescence intensity (MFI) of CD103 expression increasing throughout the time course of CS exposure, from 2 to 12 wk (P < 0.0001) (Fig. 6, E–G). CS also modestly increased CD103 expression on CD4^+^ (P = 0.0004) and CD8^+^ (P = 0.018) T cells, with both these cell types expressing much lower levels compared with MAIT cells at 12 wk of CS exposure (Fig. 7). CS exposure increased the frequency of IL-17 production after 8 wk (P = 0.0014) (Fig. 6 H), increased the frequency of PD1 expression throughout the time course of 2–12 wk (P < 0.0001) (Fig. 6 I), and reduced the frequency of CD38 expression on MAIT cells at 2, 4, and 12 wk of exposure (Fig. 6 J). In contrast, IL-17 production by CD4^+^ and CD8^+^ T cells was unaffected, while PD1 and CD38 expression were more highly induced on CD4^+^ T cells (P < 0.0001) by 12 wk of CS exposure (Fig. 7).

**Figure 8.**
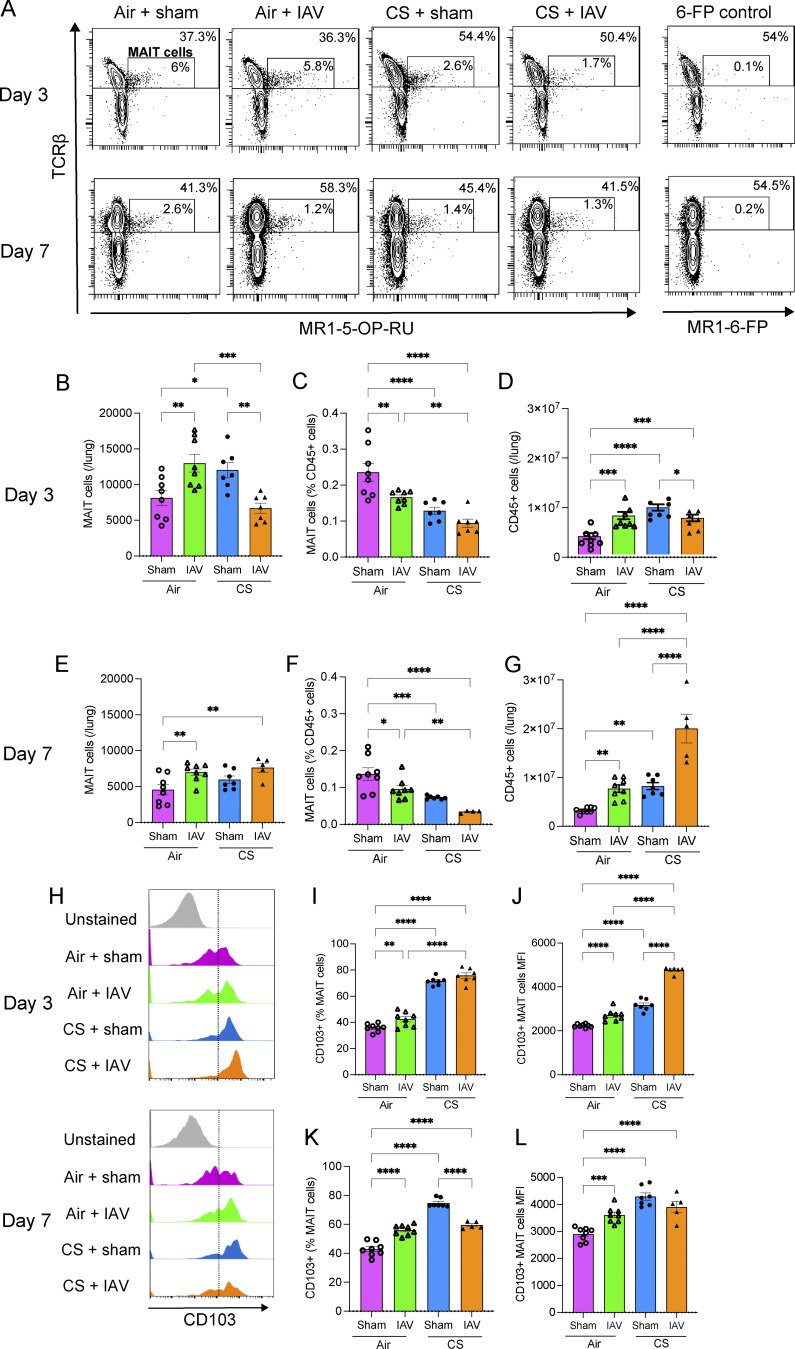
**CS exposure alters MAIT cell responses and CD103 expression during IAV infection in vivo. (A)** Representative flow cytometry plots, including MAIT cells (CD45^+^ TCRβ^+^ MR1–5-OP-RU tetramer^+^ PLZF^+^ CD44^hi^ NK1.1^−^ CD19^−^ CD11b^−^). **(B–G)** (B and E) Total numbers of MAIT cells per lung, (C and F) frequency of MAIT cells as a percentage of CD45^+^ cells, and (D and G) total numbers of CD45^+^ cells per lung, in lung homogenates from mice exposed to normal air or CS for 10 wk, followed by infection with IAV or sham infection, at day 3 (A–D) or 7 (A and E–G) after inoculation. **(H–L)** Representative flow cytometry plots showing (H) CD103 expression on MAIT cells, (I and K) frequency of CD103^+^ MAIT cells as a percentage of total MAIT cells, and (J and L) MFI of CD103 staining on MAIT cells, in lung homogenates from mice as in A. All data presented as mean ± SEM. *N* = 5–8 mice. Statistical analysis by one-way ANOVA with Fisher’s least significant difference post hoc test (* = P < 0.05, ** = P < 0.01, *** = P < 0.001, and **** = P < 0.0001).

Total MAIT cell numbers were increased in the lungs of mice infected with IAV at both 3 (P = 0.0021) (Fig. 8, A and B) and 7 dpi (P = 0.0046) (Fig. 8, A and E) and in mice exposed to CS for 10 wk (P = 0.0133 Fig. 8, A and B). However, prior CS exposure dysregulated the MAIT cell response to IAV infection, with mice exposed to CS+IAV showing a substantial decrease in MAIT cell numbers in the lung at 3 dpi, compared with air+IAV (P = 0.0002) or CS+sham inoculation (P = 0.0016) (Fig. 8, A and B). This effect was observed in early stages of infection, as by 7 dpi, mice in the CS+IAV group had similar numbers of MAIT cells in the lungs as mice in the air+IAV group (Fig. 8, A and E). Furthermore, the frequency of MAIT cells as a proportion of CD45^+^ cells was reduced by both IAV infection (P = 0.003 at 3 dpi; P = 0.0134 at 7 dpi) and 10 wk of CS exposure (P < 0.0001), with the greatest reduction observed in the CS+IAV group, particularly at 7 dpi (P < 0.0001) (Fig. 8, C and F). This is likely due to the intense infiltration and expansion of other cell types, such as neutrophils and macrophages, as indicated by bronchoalveolar lavage fluid (BALF) differential cell counts in these two inflammatory conditions (Fig. S5). This may dilute protective MAIT cell responses through pro-inflammatory responses that are deleterious in COPD exacerbations. The total numbers of CD45^+^ cells in the lungs was increased by both IAV infection (P = 0.0001 at 3 dpi; P = 0.0089 at 7 dpi) and CS exposure (P < 0.0001), with further substantial increases in CS+IAV at 7 dpi (P < 0.0001) (Fig. 8, D and G).

MAIT cell phenotype was also altered by IAV infection in vivo, with CD103 frequency and MFI increasing in IAV-infected mice at 3 and 7 dpi (Fig. 8, H–L). However, synergistic increases with CS+IAV were only observed in CD103 MFI at 3 dpi (Fig. 8 J). This indicates that CS exposure alters MAIT cell responses in vivo, increasing MAIT cell numbers and CD103, IL-17, and PD1 expression in the lung, but dysregulating MAIT cell accumulation in the early stages of IAV infection and may contribute to increased susceptibility to infection and the exacerbation of COPD.

### MAIT cell–deficient mice are protected from the development of COPD features

**Figure 9.**
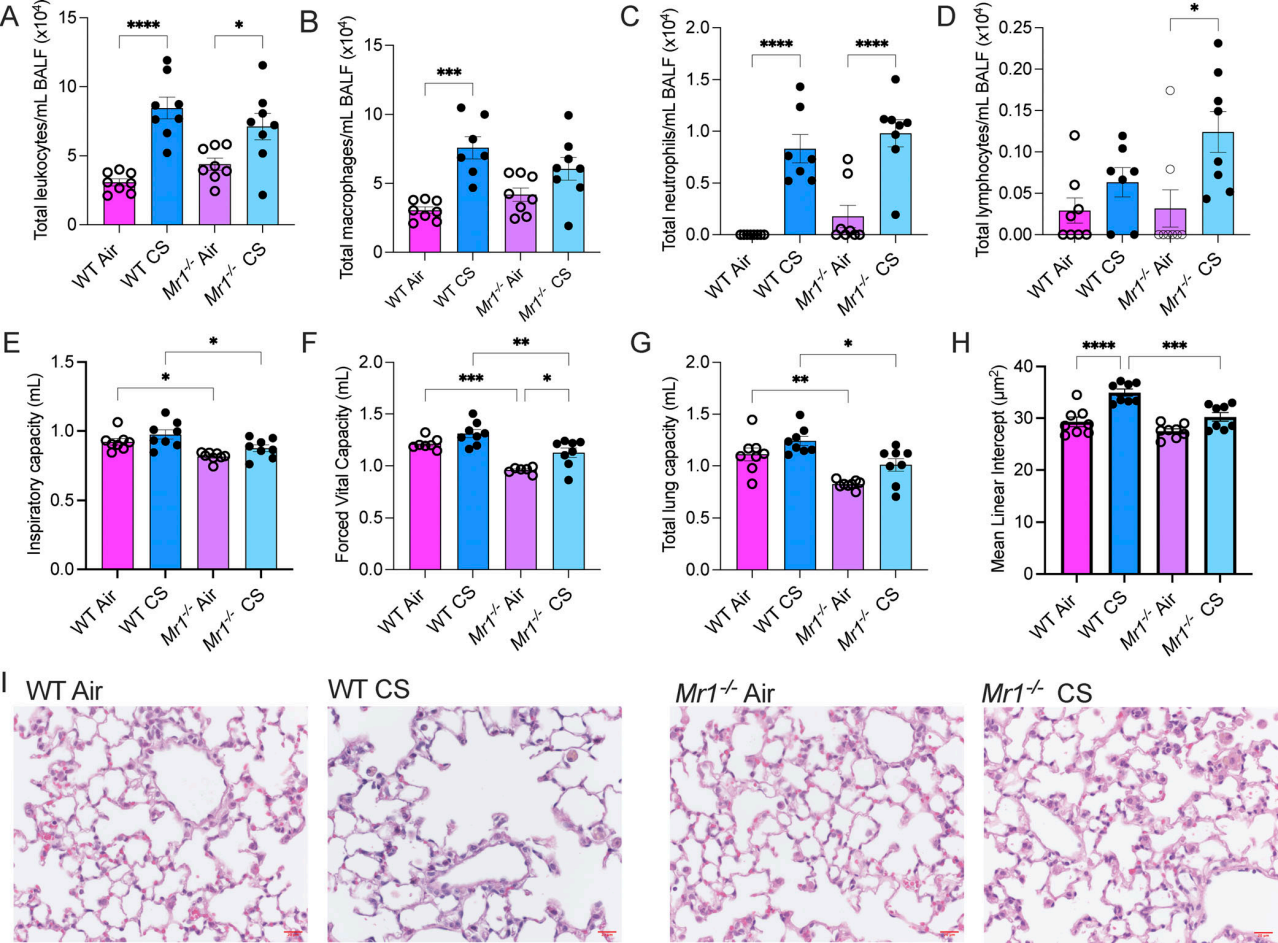
**MAIT cell deficiency is associated with protection of COPD development.** WT or MR1^−/−^ mice were exposed to room air (Air) or CS from up to 12 cigarettes, twice/day, 5 days/wk for up to 8 wk. Mice were then put through lung function perturbations and BALF collected from the single lobe of the lung. **(A–D)** Total leukocytes (A) were counted and then cytospins for differential cell counts created to quantify macrophages (B), neutrophils (C), and lymphocytes (D). **(E–H)** Lung function parameters such as inspiratory capacity (E), forced vital capacity (F), and total lung capacity (G) were measured. Histological examination of the lung parenchyma and distance between alveoli was measured to calculate emphysema (H). **(I)** Representative images of the lung parenchyma in each of the groups are shown. Scale bar represents 20 μm. Data shown are the mean ± SEM. *N* = 7–8 mice. Statistical analysis was performed using a one-way ANOVA (* = P < 0.05, ** = P < 0.01, *** = P < 0.001, and **** = P < 0.0001).

Based on our earlier findings that MAIT cell abundance in the lung was significantly elevated following CS exposure, we next determined whether MAIT cell deficiency (*Mr1*^−/−^) was associated with improved disease outcomes in a CS-exposed COPD disease model (Fig. 9). Both WT and *Mr1*^−/−^ smoke (CS) groups developed lung inflammation following CS exposure characterized by elevated BALF inflammatory cell counts, with increases in total leukocytes compared with their respective air control groups, although the increase in the *Mr1*^−/−^ group was less (Fig. 9 A). Further, we observed increases in airway macrophages in the WT CS group. However, in contrast, there was no increase in the *Mr1*^−/−^ group (Fig. 9 B). There were similar increases in neutrophils in both the WT and *Mr1*^−/−^ CS groups, with no differences between them (Fig. 9 C). There were no differences between the WT groups for lymphocytes; however, they were increased in the *Mr1*^−/−^ CS group (Fig. 9 D). Analysis of lung function parameters demonstrated that *Mr1*^−/−^ Air and CS groups had reduced inspiratory capacity (Fig. 9 E), forced vital capacity (Fig. 9 F), and total lung capacity (Fig. 9 G) compared with WT groups. We observed the development of enlarged alveoli and emphysema in the WT CS group; however, importantly, *Mr1*^−/−^ CS groups did not develop emphysema (Fig. 9, H and I). This supports our lung function data, demonstrating that MAIT cell–deficient mice were partially protected from the development of COPD disease features that were associated with CS exposure.

## Discussion

Cigarette smoking compromises the host’s ability to respond to respiratory infections and provokes inflammatory responses. It also significantly increases the risk of microbial infections in the lungs, yet it is unclear whether this is due to increased susceptibility to respiratory pathogens or exaggerated pro-inflammatory responses to microbes (Stämpfli and Anderson, 2009). Furthermore, exposure to CS typically contributes to the pathogenesis of smoking-related pathologies such as COPD (Feng et al., 2011; Lee et al., 2012; Stämpfli and Anderson, 2009). COPD is the third leading cause of chronic morbidity and death globally (WHO, 2021) and is characterized by poorly reversible airflow obstruction and abnormal inflammatory response in the lungs. However, the mechanisms that drive the development and progression of CS-induced chronic inflammation and COPD are poorly understood, and this has severely hampered the development of effective treatments. MAIT cells have recently been reported to be significantly reduced in the peripheral blood of smokers and patients with COPD, yet accumulate in the lungs of COPD patients (Kwon et al., 2016; Qiu et al., 2021). In explanation of this, there is emerging evidence that MAIT cell function is affected by CS. A recent study showed that exposure to CS decreased IFN-γ production by a MAIT cell clone in response to primary human broncho-epithelial cells infected with *Streptococcus pneumoniae* (Huber et al., 2023). Whether CS components can bind MR1, impact MAIT cell functions in the lungs, and how they are related to the pathogenesis of COPD are yet to be elucidated.

We describe a range of compounds found in CS that bind to MR1, and we detail the structural basis of their interactions, thereby revealing novel scaffolds that bind within the A′ pocket of MR1. These MR1-binding CS ligands formed Schiff base covaelent bond with MR1-K43 and mediated few interactions with MR1 residues consistent with the thermal stability of the refolded MR1–CS complexes. While the ligands identified here clearly bind to MR1, they do not stimulate MAIT cells. This is not surprising as the ability to bind MR1 is insufficient alone to confer MAIT cell stimulatory properties (Awad et al., 2020; Keller et al., 2017; Kjer-Nielsen et al., 2012). More broadly, analogous to canonical presentation of peptides by MHC molecules to T cells, where non-stimulatory ligands are the rule and activation the exception, MR1 seems to similarly bind to several small molecules without stimulating MAIT cells, as the latter could be detrimental if MAIT cells promiscuously recognize, and are activated by, a broad panel of chemical scaffolds.

Some of the CS ligands we identified do moderately inhibit MAIT cell function, both in vitro and ex vivo. Interestingly, while CSE caused mild activation of MAIT reporter cells, the combination of CSE and 5-OP-RU resulted in lower activity than 5-OP-RU alone. We consider this is likely due to competition for MR1 binding, as non-agonist MR1 ligands, such as 6-FP and Ac-6-FP, have similar effects (Keller et al., 2017; Patel et al., 2013). The possibility of negative TCR-dependent signaling has not been examined. We also found nonspecific inhibitory effects of CSE on non-MAIT T cells and on MAIT cell activation through Ag-independent mechanisms. However, while the inhibitory effects of the MR1-binding CS ligands may be considered subtle, it is important to consider this in the context of the pathogenesis of COPD. Pathogenesis is a very slow process, taking 20–40 years for COPD to develop, being driven by the gradual accumulation of small chronic effects of many inhalation exposures. Thus, our findings of components of CS that cause small perturbations in MAIT cell responses over time are consistent with the biology and pathogenesis of the disease and with our in vivo model, which clearly demonstrated the effects of CS exposure on MAIT cell function.

We identified multiple, potentially competing, effects of CS and its components on MAIT cell immune responses, which contribute to driving COPD pathogenesis and impair lung immune responses to respiratory pathogens. COPD patients are highly susceptible to influenza that exacerbates their conditions (Hsu et al., 2015; Kedzierski et al., 2017). Previous studies showed that CS exposure causes significantly reduced immune responses to IAV infection (Wu et al., 2021). Here, we show that altered MAIT cell activity is linked to the pathogenesis of COPD and its infectious exacerbations, extending the reach of the basic observations to two different and important clinical scenarios. Since we found several components to have different effects, we used mouse models of whole CS exposure to examine the impact on MAIT cells. We found that in vivo exposure to CS alters MAIT cell responses, promotes the accumulation of MAIT cells in the lung, and increases MAIT cell expression of CD103, IL-17, and PD1 expression but decreases their CD38 expression. CD103 is a mucosal-homing receptor, and increased expression on MAIT cells with CS and IAV exposure could indicate differentiation and/or expansion of a tissue-resident phenotype and increased tissue retention in these inflammatory conditions. However, CS exposure reduced the numbers of MAIT cells in the lungs during early responses to an exacerbating IAV infection, and IAV infection failed to increase the frequency of CD103 expression on MAIT cells after CS exposure. This suggests that, while CS promotes the accumulation of MAIT cells in the lungs in the absence of infection, these cells are unable to respond appropriately to pathogenic infection, and their infection-induced expansion is delayed. This dysregulation in MAIT cell responses may contribute to the development and exacerbation of smoking-associated inflammatory diseases such as COPD and to the increased susceptibility to infections and infectious exacerbations that are major clinical issues in these disorders. IL-17 facilitates neutrophilic inflammation and contributes to airway remodelling and disease progression in COPD (Lai et al., 2018; Ma et al., 2023). PD1 is a maker of T cell exhaustion that is increased on T cells from patients with COPD (Wilkinson, 2017). Chronic engagement of PD1 leads to a loss of effector function and, therefore, the ability to mount protective immunity against infections. CD38 is a T cell activation marker that is increased on MAIT cells during their response to numerous viruses and is required for antiviral responses by other cell types (Long and Hinks, 2021). Collectively, this suggests that in vivo CS increases MAIT cell influx associated with increases in their expression of the chemotactic marker CD103 but reduces their function with increases in the exhaustion marker PD1 and reduction in the activation marker CD38, hampering their ability to respond to infection appropriately. It also promotes MAIT cell production of IL-17, which may contribute to driving inflammation and the pathogenesis of COPD. Indeed, our data using *Mr1*^*−/−*^ mice show that the development of lung inflammation and altered function and emphysema were all reduced, showing that MR1 and MAIT cells are involved in the induction of the hallmark features of COPD. Our data suggest that CS attenuates MR1-dependent MAIT cell activity but also drives pro-inflammatory responses. Both of these could be inextricably linked whereby altered MAIT cell function drives inflammatory responses from other cell types and therefore COPD pathogenesis (as suggested by our *Mr1*^−/−^ data), and CS could also directly drive inflammatory responses from other cells such as macrophages that also contribute to inflammation. Furthermore CS-induced cytokines may locally activate and recruit MAIT cells in a TCR-independent manner bypassing MR1 blockade by CS compounds. These are complex issues that require extensive in vivo experiments to delineate these possibilities in future studies.

## Materials and methods

### Antibodies and staining reagents

MR1-specific 8F2.F9 and 26.5 antibodies and MHC I–specific antibody W6/32 were produced in-house from hybridomas. Commercially available antibodies and reagents used for staining are outlined in Table S3.

### Preparation of CS extract

Reference 3R4F tobacco cigarettes (University of Kentucky) with filter removed were used to create CSE (Paudel et al., 2022). Smokers smoke cigarettes and other tobacco products that do or do not have filters, so either exposure is representative of some individuals. Removing the filters ensures that we extract all of the tobacco compounds from the cigarettes; therefore, we elected to remove the filters to have more comprehensive coverage. This matches with the compounds used in in vitro studies. CSE (100%) was prepared by extracting combusted smoke from one cigarette through tubing and suction using a 50-ml syringe. Extracted smoke was dispersed through a mixing cannula into 10 ml of milliQ water in borosilicate glass vials. We attempted to resuspend CSE in several solvents, including DMSO, ethanol/chloroform, and water. However, CSE dissolved in other solvents was toxic to cells; therefore, we focused our studies on CSE extracted in water. CSE is predicted to comprise about 20,000 structurally different molecules (Fig. 2 A), making it difficult to identify and confirm the presence of the ligands revealed by our in silico research in the CSE solution using mass spectrometry methods, which could be further examined in the future. Nicotinaldehyde, a pyrolysis product of tobacco observed in third-hand smoke exposure and formed by the oxidation of nicotine deposited on indoor surfaces, is one of the key ligands that we used to show had an impact on the MR1–MAIT axis. Smoke extraction and dispersion into the same 10 ml of milliQ water was repeated until the cigarette was completely combusted. CSE samples were sterile filtered with 0.22-µm syringe filters, stored frozen at −80°C, and diluted at indicated concentrations for cell culture experiments.

### Chemicals

5-OP-RU was synthesized as a solution in DMSO and its concentration was quantified by NMR according to previously published methods. It is stable in DMSO and when bound to MR1, but it is unstable in aqueous solutions (Mak et al., 2017). Its exposure to water and moisture should be minimized during handling and dilution. Smoke component chemicals were purchased from Sigma-Aldrich and Sapphire Bioscience.

### Flow cytometry

For standard flow cytometry cell staining (unless otherwise specified), cells were stained with antibody mixtures diluted in PBS with 2–10% fetal bovine serum (Sigma-Aldrich) for 30 min on ice. Flow cytometry data were collected using FACS CantoII (BD Biosciences) or BDFortessa (BD Biosciences) flow cytometers. Data were analyzed using FlowJo cell analysis software (FlowJo, LLC).

### MR1 upregulation assays

MR1 surface expression upregulation assays were performed as previously reported (Kjer-Nielsen et al., 2012; Reantragoon et al., 2012). C1R or C1R.MR1 cells (1 × 10^5^) were incubated with 5-OP-RU, Ac-6-FP, CSE, or smoke compounds for 3 or 16 h. Cells were subsequently stained with biotinylated anti-MR1 mAb 8F2.F9 or 26.5 for 30 min on ice, followed by PE-conjugated streptavidin for MR1 expression and directly conjugated antibodies to detect HLA-A,B,C (W6/32, Alexa Fluor 700 conjugated) or CD86 (APC conjugated) as indicated. Cells were then stained with 7AAD viability dye (1:500) and fixed with 1% paraformaldehyde before flow cytometry analysis.

### Jurkat.MAIT activation and inhibition assays

MAIT reporter cell line activation assays were performed as previously reported (Kjer-Nielsen et al., 2012; Reantragoon et al., 2012). Jurkat.MAIT cells (1 × 10^5^) were co-incubated at a 1:1 ratio with C1R or CIR.MR1 cells for 3–16 h in 200 μl complete media with 5-OP-RU, Ac-6-FP, CSE, or smoke compounds. Control Jurkat.LC13 cells were activated by C1R cells expressing HLA-B8 in the presence of the control EB viral peptide FLR or smoke compounds. Cells were subsequently stained with PE-Cy7–conjugated anti-CD3 (1:300) and APC-conjugated anti-CD69 (1:50) or with CD3-APC (1:50) and CD69-PE (1:50) before flow cytometry analysis. Activation of Jurkat reporter cells was measured by increases in surface CD69 expression. IL-2 released by Jurkat.MAIT cells was assayed by indirect ELISA (using 100 μl of cell supernatants) with the conversion of o-Phenylenediamine dihydrochloride (Sigma-Aldrich) by horseradish peroxidase and measurement of absorption at 492 nm (Reantragoon et al., 2013).

### Computational methods

All molecular modelling was performed using Schrödinger Suite (v 2020-3) with the ternary crystal structure of MR1–5-OP-RU–TCR (PDB: 6PUC) from the Protein Data Bank (https://www.rcsb.org/). Water molecules were removed from the protein structure in Maestro with atom and bond types corrected, protonation states of ionizable species adjusted to pH 7.0 using Epik, and H-bond assignments optimized. Ligands were constructed as 2D molecular structures using ChemDraw, converted to 3D Structure Data File format for docking, and prepared using LigPrep module in Schrödinger. The OPLS3e force field was used and ionization states were assigned at pH 7 ± 2.0. During receptor grid generation for covalent docking, the ligand was enclosed in a box centered on 5-OP-RU from the co-crystal structure (inner box 10 Å × 10 Å × 10 Å; outer box 26 Å × 26 Å × 26 Å). The nucleophilic residue Lys43 from MR1 was set as attachment residue for covalent bond formation. “Imine Condensation” was used as reaction type during docking simulation. One pose/ligand was saved for interaction analysis. For non-covalent docking, the docking grid was similarly centered on 5-OP-RU and Glide Standard Precision mode with default setting was implemented. Ligand binding affinity was predicted using the docking score from GLIDE with covalent ligands ranking from −4.1 kcal/mol (1*H*-pyrrole-2-carbaldehyde) to −6.3 kcal/mol (α-methylcinnamaldehyde), whereas non-covalent ligands ranged from −5.1 kcal/mol (2,3-dihydroxysuccinic acid) to −6.8 kcal/mol (peconazole). Chemoinformatics analysis was performed using Canvas in Schrödinger. The “Library Comparison” module was used by finding the nearest neighbor in the reference library (19 ligands selected as putative MR1 binders) for each compound in the query library (5-OP-RU or diclofenac), using fingerprint similarity (ECFP4). Nearest neighbor similarities were returned in a column named MaxSim. Results were used to create a histogram from nearest neighbor similarities. To assess library diversity, the reference library was compared with itself.

### PBMC preparation, activation assay, and staining

Healthy adult blood donors were either from laboratory volunteers or buffy packs obtained from the Australian Red Cross Lifeblood following approval from the Monash or University of Melbourne Human Research Ethics Committees, respectively (Monash approval: 19488, University of Melbourne approval: 12540). PBMCs were isolated by gradient centrifugation using Lymphoprep (STEMCELL Technologies). Cells were then cryogenically frozen for later analysis. Activation of MAIT cells in PBMC cultures was assayed as previously described (Corbett et al., 2014). PBMCs were thawed, washed, then cultured for 2 h before adding ligand, CSE, or DMSO control and culturing for 4 h. Brefeldin A solution (eBioscience) was then added and cells cultured for a further 18 h. Cells were collected and stained with viability dye, prior to fixation and permeabilization using the eBioscience Intracellular Fixation & Permeabilization Buffer Set (Invitrogen). Antibodies diluted in permeabilization buffer were then incubated with cells for 45 min at room temperature. For tetramer titrations, PBMCs were stained with various dilutions of tetramers for 15 min at room temperature. Samples were washed and stained with antibodies (as described in the text) for 30 min on ice. PBMCs were incubated in the presence or absence of 5% CSE, 100 μM of veratraldehyde, nicotinaldehyde, salicylaldehyde, 3,4-dihydroxybenzaldehyde, penconazole, or anilazine for 1 h prior to addition of PMA (5 ng/ml) and ionomycin (1 μg/ml) to activate the cells. After a further 1-h incubation, BD Golgi plug was added, and cells were incubated for 18 h before being stained with surface antibodies (MR1–5-OP-RU tetramer-PE, CD3-PE-CF594, CD161-PE-Cy7, CD8α-PerCPCy5.5, and CD4-BUV496), then fixed with 1% paraformaldehyde, permeabilized with 0.3% saponin and stained intracellularly with antibodies to cytokines (TNF-APC and IFNγ-AF700), and analyzed by flow cytometry to determine the impact of CSE or CS components on T cell activation independent of the TCR.

### Expression, refold, and purification of MR1-β2m and MAIT TCR proteins

Human WT MR1-β2m was refolded as described previously (Corbett et al., 2014; Reantragoon et al., 2013) in the presence of Ac-6-FP, 5-OP-RU, or compounds corresponding to components of CS. Soluble A-F7 (TRAV1-2/TRBV6-1) TCR protein was refolded from inclusion bodies, as described previously (Eckle et al., 2014). Refolded MR1 ligand and TCR proteins were purified by three consecutive purification steps: crude anion exchange, S200 15/60 size-exclusion chromatography, and HiTrap-Q HP anion exchange. Protein purity was assessed using SDS-PAGE and quantified using a NanoDrop spectrophotometer.

### Thermal stability assay

A thermal shift assay of MR1-Ags was performed to monitor protein stability upon heating, using fluorescent dye SYPRO Orange in a real-time detection system (Corbett RotorGene 3000). The MR1 protein complexed with each ligand was purified by gel filtration just prior to the experiment in buffer of 10 mM Tris-HCl (pH 8) and 150 mM NaCl. Samples were then heated from 25°C to 90°C with a heating rate of 1°C/min. Fluorescence intensity was measured at excitation 530-nm and emission 610-nm wavelengths. The Tm50 represents the temperature for which 50% of the protein is unfolded. Experiments were conducted in triplicate at three independent times.

### Protein crystallization, structure determination, refinement, and analysis

Soluble MAIT A-F7 TCR was mixed with MR1-Ag proteins in a 1:1 M ratio at a concentration of 4–6 mg/ml and incubated on ice for 1 h. A hanging-drop method was used to grow crystals with a precipitant consisting of 10–18% PEG3350, 100 mM Bis-Tris-Propane (pH 6.1–6.6), and 200 mM sodium acetate, as reported previously (Awad et al., 2020). TCR–MR1–Ag complex crystals formed within 2 wk at 20°C and were flash frozen in liquid nitrogen after a short soak in reservoir solution with 10–14% glycerol for cryoprotection. X-ray diffraction data were collected at 100 K on the Australian Synchrotron at either MX1 or MX2 beamlines. Diffraction images were processed using XDS (Kabsch, 2010) and programs from the CCP4 suite (Winn et al., 2011) and Phenix package (Adams et al., 2010). TCR–MR1–Ag ternary structures were determined by molecular replacement using Phaser (McCoy, 2007) in Phenix, where modified TCR–MR1 ternary complex (PDB: 4PJ5) was used as a search model. The Grade Web Server and Phenix tools were used to build and generate ligand restraints. Model building was performed in Coot (Emsley and Cowtan, 2004), with repetitive refinement rounds using Phenix.refine (Adams et al., 2010). The models were validated using MolProbity (Chen et al., 2010) and graphical representations were generated using PyMOL Molecular Graphics System, Version 2.0 (Schrödinger, LLC).

### Murine CS exposure and IAV infection

Murine experiments were performed at the Centenary Institute, Sydney, or the Hunter Medical Research Institute, University of Newcastle, Australia, with all protocols approved by the Sydney Local Health District Animal Welfare Committee or the University of Newcastle Animal Care and Ethics Committee. Mice were housed in individually ventilated and filtered cages under positive pressure in an specific pathogen-free (SPF) facility and fed a standard mouse diet available ad libitum (Speciality Feeds). Female C57BL/6 mice (8–12 wk old) were exposed to the smoke (nose only) from 12 3R4F cigarettes, twice a day, five times a week for up to 12 wk, as described previously (Beckett et al., 2013; Donovan et al., 2019). Mice were euthanized by sodium pentobarbital (Lethabarb; Virbac) overdose at 2, 4, 6, 8, or 12 wk of CS exposure and the lungs perfused with PBS for assessment of MAIT cells. Some groups of mice were intranasally inoculated with IAV (H1N1 A/PR/8/34 mouse adapted, 33 PFU, in 50 μl UltraMDCK media) or were sham inoculated (UltraMDCK media) under isoflurane anesthesia (Kim et al., 2017) at 10 wk of CS exposure with cessation of CS exposure 1 day before infection and euthanized by sodium pentobarbital overdose at 3 or 7 days after inoculation for assessment of MAIT cells.

### *Mr1* ^*−/−*^ mice and CS exposure

Female C57BL/6 mice and female *Mr1*^*−/−*^ mice (8–12 wk old) were exposed to CS exposure for up to 8 wk, as previously described (Beckett et al., 2013). At the conclusion of the experiment, mice were placed into a state of surgical anesthesia with a combination of ketamine/xylazine (Virbac) and mice cannulated with an 18G custom cannula before being ventilated on a flexiVent lung function machine (Scireq). Mice were ventilated at 450 breaths/min and placed through a series of forced oscillation and forced manoeuvre techniques to assess lung function parameters such as inspiratory capacity, forced vital capacity, and total lung capacity (Beckett et al., 2013). Animal ethics applications are through the Sydney Local Health District Animal Welfare Committee.

### Flow cytometry of murine lung cells

Mouse MR1–5-OP-RU and MR1–6-FP tetramers were generated as previously described (Rahimpour et al., 2015). Flow cytometry was performed on single-cell suspensions from whole mouse lungs (Donovan et al., 2019; Starkey et al., 2016). The lung tissue was digested into single-cell suspensions with collagenase D (2 mg/ml; Roche) and DNase I (80 U/ml; Roche) using a GentleMACS Dissociator according to the manufacturer’s instructions (Miltenyi Biotec). Red blood cells were removed with lysis buffer (155 mM NH_4_Cl, 12 mM NaHCO_3_, and 0.1 mM ethylenediaminetetraacetic acid, pH 7.35, 5 min, 4°C). Total live-cell counts were performed using a hemocytometer. For intracellular cytokine staining, cells were stimulated with PMA (20 ng/ml) and ionomycin (0.5 µg/ml) with Brefeldin A (5 µg/ml) in complete RPMI (3 h, 37°C). Cells were stained with eBioscience Fixable Viability Dye eFluor 506 (30 min, 4°C; Thermo Fisher Scientific) then blocked using mouse Fc receptor block (anti-mouse CD16/32; 10 ng/ml; BioXcell) and unlabelled MR1–6-FP control tetramers (1:100; 15 min, 4°C). Cells were then stained with MR1–5-OP-RU–PE or MR1–6-FP–PE control tetramers and surface marker antibodies anti-CD45-PerCP, TCRβ-APC, CD4-BV711, CD8a-BV605, NK1.1-FITC, CD19-FITC, CD11b-FITC, CD44-BUV737, and CD103-BV786, with PD1-APC-Cy7 and CD38-BV650, or CD3ε-BUV395 (30 min, 4°C; BD Biosciences; Biolegend). Cells were fixed and permeabilized using eBioscience Foxp3/Transcription Factor Staining Buffer Sets (Thermo Fisher Scientific) and stained with anti-IL-17-BV421 and/or PLZF-PE-CF594 (BD Biosciences; Biolegend). Cells were then analyzed using a Fortessa X-20 flow cytometer (BD Biosciences) and FACSDiva software (BD Biosciences). After exclusion of doublets, cell debris, and dead cells, MAIT cells were identified based on forward and side scatter and characteristic antigen expression (CD45^+^ TCRβ^+^ MR1–5-OP-RU tetramer^+^ PLZF^+^ CD44^hi^ NK1.1^−^ CD19^−^ CD11b^−^; Fig. S5) (Rahimpour et al., 2015).

### Airway cellular inflammation

Differential cell counts were performed on cells from BALF to assess airway inflammation in the murine model (Beckett et al., 2013). The single left lung lobe was lavaged (2 × 500 μl PBS) via a cannula inserted into the trachea. Red blood cells were lysed and remaining leukocytes counted by trypan blue (Sigma-Aldrich) exclusion using a hemocytometer. Leukocytes were cytospun (300 × *g*, 10 min; Thermo Fisher Scientific) onto microscope slides then stained with May–Grunwald and Giemsa stain. Immune cells were discriminated and enumerated using a light microscope (40× magnification) based on key morphological characteristics (Horvat et al., 2010).

### Emphysema

Mouse lungs were perfused, inflated, and formalin-fixed and 4-μm sections of paraffin-embedded lung tissue were mounted onto slides and stained with hematoxylin and eosin. Emphysema-like alveolar enlargement was quantified using the mean linear intercept (MLI) technique, as previously described (Jarnicki et al., 2016). Briefly, a standardized template of horizontal lines was laid over randomly acquired micrographs of parenchymal tissue from lung sections (10 per mouse). The number of intercepts between alveolar walls and template lines were counted, the average number of intercepts for each mouse determined, and MLI calculated based on cumulative length of template lines and number of intercepts. Increased MLI length indicates increased alveolar size and emphysema.

### Statistics

Statistical analysis was performed using GraphPad Prism version 8 for macOS, GraphPad Software. Significance is indicated where P < 0.05. Comparisons between mouse groups were quantified using one-way ANOVA with Fisher’s least significant difference post hoc test.

### Online supplemental material

Fig. S1 shows cellular screening of compounds identified as CS components for antigen presentation. Fig. S2 shows various Jurkat.MAIT activation by smoke compounds. Fig. S3 shows activation/inhibition of MAIT cells and T cells within PBMCs by CSE, CS components. Fig. S4 shows the effect of CSE and components on TCR-independent (CD3/CD28) activation of T cells. Fig. S5 shows airway inflammation during in vivo CS exposure and IAV infection, and gating strategy for characterization of murine lung MAIT cells. Table S1 shows the summary of the tested in silico hits. Table S2 shows diffraction data collection and refinement statistics. Table S3 shows antibodies used for flow cytometry.

## Supplementary Material

Table S1 shows the summary of the tested in silico hits.

Table S2 shows diffraction data collection and refinement statistics.

Table S3 shows antibodies used for flow cytometry.

## Data Availability

The data that support the findings of this study are available from the corresponding authors upon request. The atomic coordinates and structure factors of the ternary complexes of A-F7 TCR–MR1 in complex with 3,4-dihydroxybenzaldehyde, veratraldehyde, nicotinaldehyde, and salicylaldehyde have been deposited in the Protein Data Bank (https://www.rcsb.org) with accession codes: 9BTX, 8BTY, 9BTZ, and 9BU0.
