# Supplementary material for: Cigarette smoke components modulate the MR1–MAIT axis
Source: J Exp Med. 2025 Jan 17;222(2):e20240896. doi: 10.1084/jem.20240896 (PMC11740918; doi:10.1084/jem.20240896)
Supplement: Table S1 — shows the summary of the tested in silico hits. [file jem_20240896_tables1.docx]

**Table S1. Summary of the tested *in silico* hits**

| **Name** | **MW** | **Source** |
| --- | --- | --- |
| Nicotinaldehyde | 107.11 | - Pyrolysis product from tobacco  - Major components of the thirdhand Tobacco smoke |
| 2,3-dihydroxy-benzaldehyde | 138.12 | - Pyrolysis product from tobacco |
| 3,4-dihydroxy-benzaldehyde | 138.12 | - Pyrolysis product from tobacco |
| Veratraldehyde | 166.17 | - Pyrolysis product from tobacco  - Used as flavor for both conventional and e-cigarettes |
| Salicylaldehyde | 122.12 | - Pyrolysis product from tobacco |
| 3-Furaldehyde | 96.1 | - Pyrolysis product from tobacco |
| 5-(hydroxymethyl)furan-2-carbaldehyde | 126.11 | - Pyrolysis product from tobacco’s cellulose |
| Syringic acid | 198.2 | - Phenolic component of the tobacco plant |
| 1H-Pyrrole-2-carboxaldehyde | 95.1 | - Derived from the roots and leaves of the tobacco |
| 2,3-Dihydroxybutanedioic acid | 150.09 | - Cigarette ingredient as appetite-suppressant additives |
| 2-Acetylpyridine | 121.10 | - Cigarette ingredient as appetite-suppressant additives |
| 4-methoxybenzaldehyde | 136.15 | - Flavor for both conventional and e-cigarettes. |
| α-Methylcinnamaldehyde | 146.19 | - Tobacco flavor |
| 3-Acetylpyridine | 121.10 | - Tobacco flavor |
| 2-Acetyl-3-methylpyrazine | 136.20 | - Tobacco flavor |
| 2-Acetylpyrrole | 109.10 | - Tobacco flavor |
| 5-Methyl-2-thiophenecarboxaldehyde | 126.20 | - Tobacco flavor |
| Penconazole | 284.2 | - Pesticides for tobacco plants |
| Anilazine | 275.52 | - Pesticides for tobacco plants |
