# Supplementary material for: Cigarette smoke components modulate the MR1–MAIT axis
Source: J Exp Med. 2025 Jan 17;222(2):e20240896. doi: 10.1084/jem.20240896 (PMC11740918; doi:10.1084/jem.20240896)
Supplement: Table S2 — shows diffraction data collection and refinement statistics. [file jem_20240896_tables2.docx]

**Table S2. Diffraction data collection and refinement statistics**

|  | **TCR-MR1-Veratraldehyde** | **TCR-MR1-Nicotinaldehyde** | **TCR-MR1-Salicyladehyde** | **TCR-MR1-3,4-dihydroxybenzaldehyde** |
| --- | --- | --- | --- | --- |
| **Wavelength (Å)** | 0.9537 | 0.9537 | 0.9322 | 0.9536 |
| **Resolution range (Å)** | 39.91 - 2.85 (2.955 - 2.85) | 49.84 - 3.00 (3.11 - 3.00) | 48.27 - 2.89 (2.99 - 2.89) | 36.19 - 2.05 (2.12 - 2.05) |
| **Space group** | C 1 2 1 | C 1 2 1 | C 1 2 1 | C 1 2 1 |
| **Unit cell**  **a, b, c (Å)**  **α, β, γ (°)** | 216.84 70.28 143.21 90 104.12 90 | 214.57 69.62 143.34 90 104.17 90 | 217.09 71.19 144.39 90 104.76 90 | 217.13 70.41 143.57 90 104.61 90 |
| **Total reflections** | 222966 (20545) | 169305 (15947) | 148434 (14575) | 427544 (40598) |
| **Unique reflections** | 48417 (4528) | 40821 (4039) | 47350 (4685) | 128700 (12714) |
| **Multiplicity** | 4.6 (4.5) | 4.1 (3.9) | 3.1 (3.1) | 3.3 (3.2) |
| **Completeness (%)** | 98.36 (92.44) | 97.83 (96.08) | 98.10 (98.09) | 97.48 (97.06) |
| **Mean I/sigma(I)** | 11.65 (2.45) | 9.06 (2.26) | 10.47 (3.86) | 11.71 (1.47) |
| **Wilson B-factor** | 56.05 | 52.32 | 40.39 | 43.21 |
| **R-merge** | 0.124 (0.7572) | 0.1411 (0.5403) | 0.1684 (0.3177) | 0.05743 (0.7641) |
| **R-pim** | 0.06374 (0.3897) | 0.07967 (0.311) | 0.1297 (0.2099) | 0.03714 (0.5066) |
| **CC1/2** | 0.995 (0.772) | 0.99 (0.851) | 0.879 (0.866) | 0.998 (0.772) |
| **CC*** | 0.999 (0.933) | 0.998 (0.959) | 0.967 (0.964) | 1 (0.933) |
| **R-work** | 0.1632 (0.2858) | 0.2018 (0.2381) | 0.2081 (0.2702) | 0.1860 (0.3461) |
| **R-free** | 0.2225 (0.3341) | 0.2556 (0.2984) | 0.2925 (0.3785) | 0.2191 (0.3845) |
| **Non-hydrogen atoms** | 12814 | 12800 | 12606 | 13770 |
| **macromolecules** | 12567 | 12542 | 12448 | 12853 |
| **ligands** | 56 | 71 | 50 | 84 |
| **solvent** | 191 | 187 | 108 | 833 |
| **Protein residues** | 1602 | 1599 | 1581 | 1601 |
| **RMS (bonds) (Å)** | 0.008 | 0.008 | 0.008 | 0.003 |
| **RMS (angles) (°)** | 0.97 | 1.01 | 1.00 | 0.60 |
| **Ramachandran favored (%)** | 95.56 | 95.61 | 94.64 | 98.29 |
| **Ramachandran allowed (%)** | 4.12 | 4.13 | 4.91 | 1.46 |
| **Ramachandran outliers (%)** | 0.32 | 0.25 | 0.45 | 0.25 |
| **Average B-factor** | 51.95 | 53.09 | 37.87 | 54.27 |
| **macromolecules** | 52.03 | 53.29 | 37.95 | 54.35 |
| **ligands** | 57.31 | 47.85 | 33.48 | 53.09 |
| **solvent** | 45.02 | 41.07 | 29.86 | 53.17 |

Statistics for the highest-resolution shell are shown in parentheses.
