# Supplementary material for: Cigarette smoke components modulate the MR1–MAIT axis
Source: J Exp Med. 2025 Jan 17;222(2):e20240896. doi: 10.1084/jem.20240896 (PMC11740918; doi:10.1084/jem.20240896)
Supplement: Table S3 — shows antibodies used for flow cytometry. [file jem_20240896_tables3.docx]

**Table S3. Antibodies used for flow cytometry**

| **Target Species** | **Antigen** | **Fluorochrome** | **Cat #** | **Manufacturer** | **Clone** |
| --- | --- | --- | --- | --- | --- |
| Mouse | PLZF | PE-CF594 | 565738 | BD | R17-809 |
| Mouse | CD45 | PerCP | 103130 | Biolegend | 30-F11 |
| Mouse | TCRβ | APC | 109212 | Biolegend | H57-597 |
| Mouse | CD3ε | BUV395 | 563565 | BD | 145-2C11 |
| Mouse | CD4 | BV711 | 100557 | Biolegend | RM4-5 |
| Mouse | CD8a | BV605 | 563152 | BD | 53-6.7 |
| Mouse | NK1.1 | FITC | 108706 | Biolegend | PK136 |
| Mouse | CD19 | FITC | 115506 | Biolegend | 6D5 |
| Mouse | CD11b | FITC | 101206 | Biolegend | M1/70 |
| Mouse | CD44 | BUV737 | 564392 | BD | IM7 |
| Mouse | CD103 | BV786 | 564322 | BD | M290 |
| Mouse | PD1 | APC-Cy7 | 135223 | Biolegend | 29F.1A12 |
| Mouse | CD38 | BV650 | 740489 | BD | 90/CD38 |
| Mouse | IL-17 | BV421 | 506926 | Biolegend | TC11-18H10.1 |
| Rat IgG1 | Isotype control | BV421 | 400439 | Biolegend | RTK2071 |
| Human | CD3 | BUV395 |  | BD | NCAM16.2 |
| Human | CD8 | BUV395 |  | BD | RPA-T8 |
| Human | IFN-γ | BV786 |  | BD | 4S.B3 |
| Human | TNF | PerCP-Cy5.5 |  | BD | MAb11 |
| Human | CD4 | Alexa Fluor® 700 |  | Biolegend | SK3 |
| Human | CD161 | Alexa Fluor® 700 |  | Biolegend | HP-3G10 |
| Human | CD161 | APC |  | Biolegend | HP-3G10 |
| Human | TCR Vα7.2 | BV605 |  | Biolegend | 3C10 |
| Human | CD69 | APC-Cy7 |  | Biolegend | FN50 |
| Human | CD3 | PE-Cy7 |  | Biolegend | UCHT-1 |
| N/A | Streptavidin | PE |  |  | N/A |
| N/A | eBioscience™ Fixable Viability Dye | eFluor 506 | 65-0866-18 | Life Technologies | N/A |
| N/A | LIVE/DEAD® Fixable Dead Cell Stain Kit | Aqua |  | Life Technologies | N/A |
